# Antibiofilm Activity of Essential Oils Against *Escherichia coli* on Lettuce Leaf Surfaces

**DOI:** 10.3390/foods15173040

**Published:** 2026-08-28

**Authors:** Ana Varga, Dragana Plavšić, Zorica Tomičić, Olja Todorić, Ružica Tomičić, Milica Aćimović, Lato Pezo

**Affiliations:** 1 Institute of Food Technology in Novi Sad, University of Novi Sad, Bulevar cara Lazara 1, 21000 Novi Sad, Serbia; dragana.plavsic@fins.uns.ac.rs (D.P.); olja.todoric@fins.uns.ac.rs (O.T.); 2 Faculty of Technology, University of Novi Sad, Bulevar cara Lazara 1, 21000 Novi Sad, Serbia; ruzica.tomicic@uns.ac.rs; 3 Institute of Field and Vegetable Crops, University of Novi Sad, Maksima Gorkog 30, 21000 Novi Sad, Serbia; acimovicbabicmilica@gmail.com; 4 Institute of General and Physical Chemistry, University of Belgrade, Studentski Trg 12/V, 11000 Belgrade, Serbia; latopezo@yahoo.co.uk

**Keywords:** *Escherichia coli*, adhesion, biofilm formation, essential oils, lettuce leaves

## Abstract

Background: Fresh leafy vegetables are widely consumed because of their high nutritional value but are also recognized as important vehicles for foodborne pathogens. *Escherichia coli* readily attaches to lettuce surfaces and forms biofilms, enhancing bacterial persistence and reducing the effectiveness of conventional washing procedures. This study evaluated the antibiofilm activity of dill, basil, winter savory, and peppermint essential oils (EOs) against *E. coli* during initial bacterial attachment and against preformed biofilms on polystyrene surface and lettuce leaves. Methods: Biofilm formation was assessed using Congo Red agar, the crystal violet microtiter plate assay, and a bacterial attachment assay on lettuce leaves, while biofilm architecture and the effects of EO treatment were examined by scanning electron microscopy (SEM). Antibacterial activity was determined by the broth microdilution method, and antibiofilm activity on initial bacterial attachment and preformed biofilms was evaluated at 0.5 × MIC, MIC, and 2 × MIC using the crystal violet assay and bacterial attachment assay on lettuce leaves, respectively. Results: The examined *E. coli* isolates predominantly exhibited the rdar morphotype and produced stronger biofilms at 25 °C than at 37 °C. All tested EOs significantly reduced initial bacterial attachment and disrupted preformed biofilms in a concentration-dependent manner. Dill EO showed the strongest antibacterial and antibiofilm effects, followed by basil, winter savory, and peppermint EOs. At 2 × MIC, the tested EOs reduced initial bacterial attachment by 74.17–84.63%, while dill EO reduced preformed biofilm on polystyrene by 59.90% after 60 min. SEM analysis confirmed extensive disruption of biofilm architecture and pronounced morphological damage to bacterial cells following EO treatment. Conclusions: These findings demonstrate that essential oils, particularly dill EO, effectively inhibit both the establishment and persistence of *E. coli* biofilms on polystyrene and lettuce leaves, highlighting their potential as natural antibiofilm agents for improving the microbiological safety of fresh produce.

## 1. Introduction

Fresh and minimally processed vegetables, particularly leafy greens such as lettuce, are widely consumed because of their high nutritional value and health-promoting properties. They provide important sources of vitamins, minerals, dietary fiber, and a wide range of bioactive compounds, including phenolic compounds, flavonoids, and terpenoids, that contribute to antioxidant activity and the prevention of chronic diseases [1]. However, their consumption in raw or minimally processed form increases the risk of exposure to foodborne pathogens, making fresh produce a significant concern in food safety [2].

Microbial contamination of lettuce may occur throughout the entire production chain, including pre-harvest stages through soil, irrigation water, organic fertilizers, and fecal contamination, as well as during post-harvest handling, processing, storage, and distribution [3,4]. Among foodborne pathogens, *Escherichia coli* is of particular concern because of its frequent association with outbreaks linked to fresh produce and its ability to survive on plant surfaces. Since leafy vegetables are generally consumed without heat treatment, contamination with pathogenic *E. coli* represents a significant public health concern. Epidemiological surveillance identified 78 foodborne outbreaks associated with leafy greens between 2014 and 2021, with lettuce frequently implicated as the food vehicle [5,6]. The persistence of *E. coli* on lettuce is largely attributed to its ability to adapt to the phyllosphere environment. Following attachment to leaf epidermal structures, bacterial cells modulate the expression of genes involved in adhesion, stress tolerance, and biofilm formation, thereby enhancing their survival, persistence, and colonization of plant surfaces [7].

Biofilm formation is recognized as one of the most effective bacterial survival strategies in food-related environments. Biofilms are structured microbial communities enclosed within a self-produced extracellular polymeric substance (EPS) matrix and attached to biotic or abiotic surfaces. Within these complex structures, bacterial cells exhibit enhanced tolerance to antimicrobial agents and environmental stresses through multiple mechanisms, including restricted sanitizer penetration, altered metabolic activity, the presence of persister cells, and increased horizontal gene transfer [8,9,10]. On lettuce surfaces, natural anatomical features such as stomata, crevices, and hydrophobic regions provide protected niches that facilitate bacterial attachment, colonization, and biofilm development, thereby increasing the persistence of foodborne pathogens and complicating their removal [11].

To mitigate microbial contamination of fresh produce, a variety of thermal and non-thermal decontamination strategies have been explored. Thermal treatments, including hot water, steam, and heated sanitizing solutions, are generally unsuitable for leafy vegetables because they can adversely affect texture, color, sensory attributes, and nutritional quality. Consequently, considerable attention has been directed toward non-thermal technologies such as conventional washing, high hydrostatic pressure, pulsed electric fields, ultrasound, irradiation, and chemical sanitizers including chlorine-based compounds, hydrogen peroxide, ozone, and organic acids [12,13]. Despite their potential, these interventions often face limitations related to high operational costs, reduced consumer acceptance, the formation of undesirable residues or by-products, and limited efficacy against biofilm-associated cells. Importantly, none of the currently available decontamination approaches can reliably achieve complete elimination of pathogens embedded within biofilms, highlighting the need for more effective and sustainable control strategies [14,15].

The limitations of currently available decontamination technologies, particularly against biofilm-associated pathogens, have driven the search for novel, effective, and sustainable antimicrobial strategies. At the same time, increasing consumer demand for minimally processed foods with reduced reliance on synthetic preservatives has intensified interest in natural antimicrobial compounds [16,17]. Among these, essential oils (EOs) derived from aromatic and medicinal plants have emerged as promising candidates owing to their broad-spectrum antimicrobial activity and their Generally Recognized As Safe (GRAS) status [18]. EOs are complex mixtures of bioactive compounds, including terpenes, phenolic compounds, and aldehydes, which exert antimicrobial effects through multiple mechanisms, such as disruption of bacterial cell membranes, interference with cellular metabolism and quorum sensing systems, and inhibition of biofilm formation. Numerous studies have demonstrated the efficacy of EOs against a wide range of foodborne pathogens and spoilage microorganisms, highlighting their potential as natural preservatives and decontamination agents for minimally processed foods, including fresh vegetables [19,20].

Although the antimicrobial activity of essential oils has been extensively documented, their ability to control *E. coli* biofilms on fresh produce surfaces remains poorly understood. In particular, limited information is available regarding the efficacy of EOs against biofilms formed on lettuce leaves, a complex food matrix that closely reflects real-world contamination scenarios. Therefore, this study aimed to investigate the antibiofilm activity of selected EOs against *E. coli* on lettuce leaf surfaces and to explore their potential application as natural decontamination agents for improving the microbiological safety of fresh and minimally processed vegetables.

## 2. Materials and Methods

### 2.1. Bacterial Strains and Inoculum Preparation

In accordance with the aims of this study, 24 isolates of *E. coli* obtained from food samples of animal origin were examined. The strains were obtained from the Scientific Veterinary Institute of Novi Sad, Serbia. All isolates were cultured on Tryptone Soya Agar (TSA, HiMedia Laboratories Pvt. Ltd., Mumbai, India) and incubated at 37 °C for 24 h. For inoculum preparation, three to four well-isolated colonies from TSA plates were inoculated into 5 mL of Tryptone Soya Broth (TSB, HiMedia Laboratories Pvt. Ltd., Mumbai, India) and incubated at 37 °C for 18 h. After incubation, the bacterial suspensions were homogenized and diluted 1:40 in fresh TSB. The optical density of the suspensions used for biofilm formation was adjusted to match the 0.5 McFarland standard (approximately 1 × 10^8^ CFU/mL) using a densitometer DEN-1 (Biosan, Riga, Latvia). To identify strong biofilm-producing isolates, preliminary screening was performed based on colony morphology and biofilm-forming ability. Based on the results of these preliminary assays, the isolate exhibiting the strongest biofilm-forming capacity was selected for subsequent experiments evaluating the antibacterial and antibiofilm activity of essential oils.

### 2.2. Colony Morphology Identification

The colony morphology of the tested *E. coli* isolates was evaluated according to the methods described by Solomon et al., 2005 [21] and Lu et al., 2011 [22]. The method was based on surface inoculation of salt-free Luria-Bertrani Agar (LBA, Lab M, International Diagnostics Group Plc, Bury, Lancashire, UK) supplemented with two indicator diazo dyes, Congo red (40 µg/mL) (Sigma-Aldrich, Saint Louis, MO, USA) and Coomassie brilliant blue (20 µg/mL) (Sigma-Aldrich, Saint Louis, MO, USA)-Congo Red LB agar *w*/*o* NaCl. The incorporation of these indicator dyes into the LBA medium enabled the identification of the major biofilm matrix components, namely cellulose and fimbriae. Congo red binds to cellulose and fimbriae, while Coomassie brilliant blue binds to the protein components of the biofilm matrix, primarily fimbriae. The center of each previously prepared LBA in a Petri plate (Ø 90 mm, NOEX, Komorniki, Poland) was inoculated with a single injection using a portion of a colony of the tested microorganisms previously cultivated on Nutrient Agar (NA, HiMedia Laboratories Pvt. Ltd., Mumbai, India) at 37 °C for 24 h. Colony morphology was determined after 96 h incubation at 25 °C and 37 °C, according to the phenotypic characteristics previously described by Bokranz et al., 2005 [23] and Römling et al., 2005 [24]: rdar (red, dry and rough-violet colony expresses curli fimbriae and cellulose), pdar (pink, dry and rough-pink colony, expresses cellulose), bdar (brown, dry and rough-brown colony, expresses curli fimbriae) and saw (smooth and white, no expression of curli fimbriae nor cellulose). Less-pronounced phenotypes were reported as ras (red and smooth, expresses curli fimbriae), bas (brown and smooth, expresses curli fimbriae) or pas (pink and smooth, expresses cellulose). The colony morphology assay was performed in triplicate for each *E. coli* strain tested.

### 2.3. Plant Material and Extraction

Dill (*Anethum graveolens* L.), peppermint (*Mentha piperita* L.), basil (*Ocimum basilicum* L.) and winter savory (*Satureja montana* L.) used in this study were cultivated at the Institute of Field and Vegetable Crops Novi Sad (IFVCNS), Serbia. The corresponding voucher specimens were assigned the numbers 2-1440, 2-1550, 2-1441 and 2-1561, respectively. The voucher specimens were authenticated and deposited in the Herbarium of the Department of Biology and Ecology (BUNS Herbarium), Faculty of Sciences, University of Novi Sad.

The aerial parts of dill, peppermint, basil and winter savory were collected in full flowering, whereas the fruits of dill were collected at full maturity. Essential oils were obtained by hydrodistillation using a Clevenger-type apparatus (ATICO Medical Pvt., Ambala, India) to extract EOs according to the procedure described in the European Pharmacopoeia (2004) [25]. Identification of EO components was performed by gas chromatography–mass spectrometry (GC–MS) following the methodology described by Varga et al., 2019 [26].

### 2.4. Determination of the Minimum Inhibitory Concentration of EOs Against E. coli

The minimum inhibitory concentration (MIC) of each EO against planktonic *E. coli* cells was determined according to the guidelines of the Clinical and Laboratory Standards Institute (CLSI, 2012) [27], with slight modifications. Serial two-fold dilutions of the tested essential oils were prepared in TSB using sterile 96-well microtiter plates, resulting in final concentrations ranging from 0.44 to 227.20 µL/mL. To facilitate the dispersion of the hydrophobic essential oils in the broth medium, Tween 80 (0.5%, Polyoxyethylenesorbitan monooleate, HiMedia Laboratories Pvt. Ltd., Mumbai, India) was added as a nonionic emulsifying agent before preparing the dilutions.

Each well was inoculated with 100 μL of a bacterial suspension adjusted to a final concentration of 1 × 10^6^ CFU/mL, followed by incubation at 37 °C for 24 h. Wells containing TSB with bacterial inoculum but without antimicrobial agents served as positive controls, while wells containing sterile TSB alone were used as negative controls. Additional control wells containing Tween 80 at the corresponding concentration, without essential oils, were included to verify that the surfactant itself did not exhibit antibacterial activity. All assays were carried out in three independent replicates. The MIC was defined as the lowest concentration of EO capable of inhibiting visible bacterial growth. Bacterial growth inhibition was assessed using the resazurin indicator assay, where the MIC corresponded to the lowest EO concentration that prevented the reduction of resazurin, indicating the absence of viable bacterial cells.

### 2.5. Biofilm Formation Assay

The biofilm formation assay and biofilm biomass quantification were performed using the crystal violet (CV) microtiter plate method described by Stepanović et al., 2000 [28] and Tomičić et al., 2023 [14], with slight modifications. Sterile flat-bottom 96-well polystyrene microtiter plates (Greiner Bio-One, Merck KGaA, Darmstadt, Germany) were used throughout the study. For biofilm formation, each well contained a final volume of 200 µL consisting of 180 µL of TSB and 20 µL of bacterial suspension prepared as described in Section 2.1. Negative control wells contained 200 µL of TSB only. Unless otherwise stated, plates were incubated statically for 48 h at 25 °C. The assay performed at 37 °C was carried out using the same procedure.

Following incubation, the culture medium was discarded and the wells were washed three times with 250 µL of sterile distilled water to remove non-adherent cells. The plates were air dried for 30 min in a laminar flow cabinet (Aura mini, BIOAIR, Siciano, Italy) followed by staining the wells with 250 µL of 0.5% crystal violet solution (AppliChem, Darmstadt, Germany) for 20 min at room temperature. Excess stain was removed by washing the wells six times with sterile distilled water, after which the plates were air dried for 1 h. The bound crystal violet was solubilized with 250 µL of 33% acetic acid (Zorka Pharma-Hemija, Šabac, Serbia) for 15 min, and the absorbance was measured at 630 nm using a microplate reader (Thermo Electron Corporation, Shanghai, China).

Biofilm-forming ability was classified according to Stepanović et al., 2000 [28]. The cut-off optical density (ODc) was defined as three standard deviations above the mean OD630 of the negative control. Based on the obtained OD values, isolates were classified as non-biofilm producers (OD ≤ ODc), weak (ODc < OD ≤ 2 × ODc), moderate (2 × ODc < OD ≤ 4 × ODc), or strong biofilm producers (OD > 4 × ODc). All experiments were performed in triplicate. Among the tested isolates, the strongest biofilm-producing isolate of *E. coli* was selected as the representative strain for subsequent essential oil treatment assays.

### 2.6. Effect of Essential Oils on Initial Bacterial Attachment

The effect of essential oils on the initial stages of bacterial attachment and subsequent biofilm formation was evaluated by adding the EOs at the beginning of the biofilm assay described in Section 2.5 [29]. EO solutions were prepared in TSB supplemented with 0.5% Tween^®^ 80 (Polyoxyethylenesorbitan monooleate, HiMedia Laboratories Pvt. Ltd., Mumbai, India) and tested at concentrations corresponding to 0.5 × MIC, 1 × MIC, and 2 × MIC. Each well contained 20 µL of EO solution, 160 µL of TSB, and 20 µL of bacterial suspension (approximately 10^8^ CFU/mL), resulting in a final volume of 200 µL. Positive controls consisted of 180 µL TSB and 20 µL bacterial suspension, whereas negative controls contained 200 µL TSB only. Following static incubation for 48 h at 25 °C, biofilm biomass was quantified using the crystal violet assay described in Section 2.5. The minimum biofilm inhibition concentration (MBIC_50_) was defined as the lowest EO concentration that reduced biofilm formation by at least 50% compared with the untreated control.

### 2.7. Effect of Essential Oils on Preformed Biofilm

Preformed biofilms were established according to the procedure described in Section 2.5 by incubating bacterial cultures under static conditions for 48 h at 25 °C. After removal of the culture medium and washing three times with sterile distilled water, 200 µL of EO solutions at concentrations corresponding to 0.5 × MIC, 1 × MIC, and 2 × MIC were added to the wells. The plates were incubated at room temperature for 15, 30, or 60 min. Untreated biofilms served as positive controls, while wells containing only TSB served as negative controls.

Following treatment, the EO solutions were removed, the wells were washed three times with sterile distilled water, and the remaining biofilm biomass was quantified using the biofilm assay described in Section 2.5.

Biofilm eradication was calculated according to Equation (1):

(1) Biofilm eradication (%)= (Ac−As)/Ac×100

where Ac represents the absorbance of the untreated control and As the absorbance of the EO-treated sample.

The minimum biofilm eradication concentration (MBEC_50_) was defined as the lowest EO concentration capable of reducing the preformed biofilm biomass by at least 50%.

### 2.8. Preparation of Lettuce Leaf Pieces

Lettuce (*Lactuca sativa* L.) of the domestic variety Novosadska majska maslena was used in this study. Lettuce leaf coupons were prepared according to the method described by Berger et al., 2009 [30], with slight modifications. Whole lettuce heads were purchased from local markets on the day of the experiments. The outer and visibly damaged leaves were removed and discarded. Prior to use, the selected lettuce leaves were washed with running chlorinated water to remove possible mechanical impurities, and subsequently rinsed with sterile distilled water.

The prepared leaves were placed in open Petri plates (diameter of 140 mm) and exposed to ultraviolet (UV) irradiation in a laminar flow chamber (Aura mini, BIOAIR, Siciano, Italy) for 30 min under airflow conditions to reduce the naturally occurring epiphytic microflora and remove residual surface moisture. Subsequently, leaf pieces measuring 10 × 10 mm (hereafter referred to as coupons) were aseptically excised using a sterile scalpel.

To verify the effectiveness of the decontamination procedure, one coupon was aseptically transferred into a test tube containing 1 mL of saline peptone solution (SPS) and vortexed (VELP Scientifica, Usmate Velate, Italy) at maximum speed for 1 min. The resulting suspension was transferred to 9 mL of SPS, and the number of residual microorganisms was determined using the standard plate count method on Plate Count Agar (PCA; HiMedia Laboratories Pvt. Ltd., Mumbai, India). The analysis was performed in triplicate, and the results were expressed as colony-forming units per square centimetre (CFU/cm^2^).

### 2.9. Bacterial Attachment Assay on the Lettuce Leaf Surface

Bacterial attachment to the lettuce leaf surface was evaluated according to the method described by Patel and Sharma, 2010 [31], with minor modifications. Prepared lettuce leaf coupons were placed individually into flat-bottom sterile polystyrene 12-well microtiter plates (Greiner Bio-One, Merck KGaA, Darmstadt, Germany), and inoculated with 1 mL of an *E. coli* suspension (approximately 1 × 10^8^ CFU/mL). The plates were incubated statically for 3 h at 25 °C to allow bacterial attachment. Following incubation, the coupons were rinsed three times with 2.5 mL of sterile distilled water to remove non-adherent cells. To determine the number of attached cells, each coupon was aseptically transferred into a tube containing 1 mL of sterile SPS. The procedure for determining the number of attached cells was carried out by exposing the test tubes to low-energy ultrasound of 40 kHz frequency for 1 min in an ultrasonic bath (ATM 40-3LCD, ATU, Valencia, Spain), followed by vortexing for 1 min (40 Hz). After treatment, the contents of the tubes were then resuspended in 9 mL of SPS. The number of adhered cells was determined by the standard colony counting technique on TSA, and the obtained result was expressed as log CFU/cm^2^. The ability to adhere to the leaf surface of vegetables was also tested at a temperature of 37 °C.

### 2.10. Effect of EOs on Preformed Biofilm on Lettuce Leaves

The effect of EOs on preformed biofilms was evaluated according to Trevisan et al., 2018 [32], with modifications. Biofilms were developed on lettuce leaf coupons by incubating the inoculated coupons for 24 h at 25 °C. The coupons were then rinsed three times with sterile distilled water and transferred aseptically to new sterile 12-well microtiter plates containing EO solutions at 0.5 × MIC, 1 × MIC, or 2 × MIC. Following 1 h exposure at room temperature, the coupons were rinsed again to remove residual EO and detached cells. Untreated biofilm-covered coupons served as the positive control. Three independent experiments were performed, with three coupons analyzed for each EO concentration.

Biofilm-associated cells were recovered and enumerated as described for the bacterial attachment assay (Section 2.9). Results were expressed as log CFU/cm^2^ of lettuce leaf surface, and the antibiofilm efficacy of EOs was determined by comparison with the untreated control.

### 2.11. Scanning Electron Microscopy (SEM) Analysis

Scanning electron microscopy (SEM) was used to visualize *E. coli* biofilm formation on lettuce leaf surfaces and to evaluate the structural changes in preformed biofilms following treatment with essential oils (EOs).

Lettuce leaf coupons were placed individually into sterile flat-bottom 12-well polystyrene microtiter plates, inoculated with 1 mL of an *E. coli* suspension (approximately 1 × 10^8^ CFU/mL), and incubated statically for 24 h at 25 °C. Biofilm formation was also evaluated at 37 °C. To assess the effect of EOs, preformed biofilms were treated with EO solutions as described above. After incubation or EO treatment, the coupons were rinsed three times with 2.5 mL of sterile distilled water to remove non-adherent cells.

Sample preparation for SEM was performed according to Pathan et al., 2008 [33]. The biofilms were fixed in 3% glutaraldehyde (Roth, Karlsruhe, Germany) prepared in 0.1 M phosphate buffer for 18 h at 5 °C, followed by two washes with sterile distilled water. The samples were dehydrated through a graded ethanol series (30%, 50%, 60%, 70%, and 90%) for 5 min at each concentration, followed by two immersions in 96% ethanol for 10 min each. The dehydrated samples were dried using a critical point dryer (CPD 030, BAL-TEC, Balzers, Liechtenstein), sputter-coated with gold (SCD 005, BAL-TEC; 30 mA, 90 s), and examined using a scanning electron microscope (JMS SEM-6460LV, JEOL Ltd., Tokyo, Japan) operated at an accelerating voltage of 25 kV.

### 2.12. Statistical Analysis

The experimental results were analyzed using R software (version 4.3.2). Analysis of variance (ANOVA) based on a linear mixed-effects model was conducted to evaluate the significance (*p* ≤ 0.05) of differences among groups. Post hoc comparisons were performed using Tukey-adjusted pairwise comparisons. Results are presented as mean values with standard deviation (SD).

## 3. Results

### 3.1. Biofilm Production

#### 3.1.1. Colony Morphology

**Figure 1:**
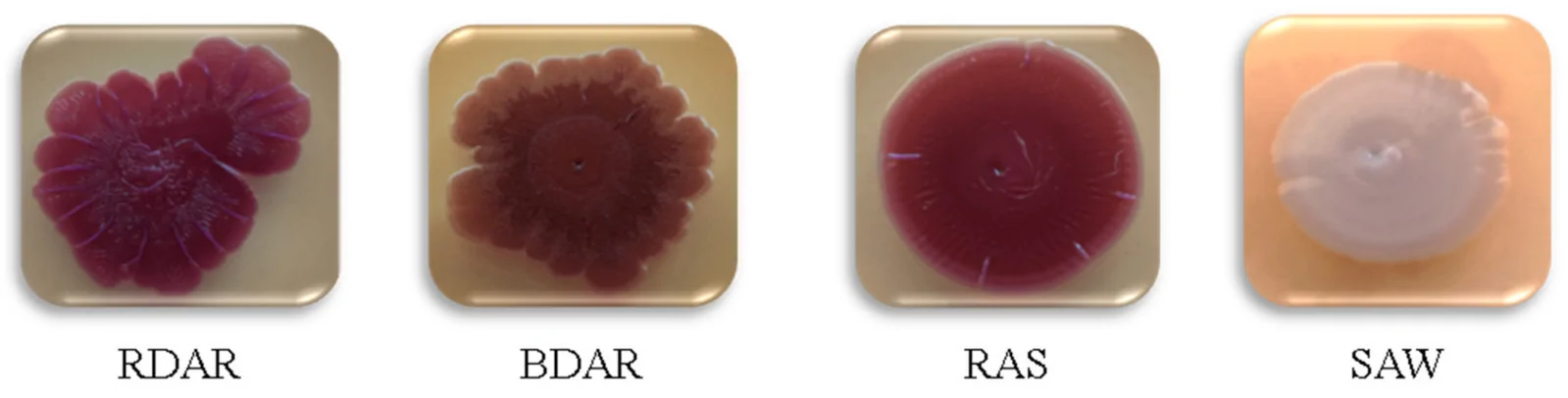
Morphotypes of *E. coli* after 96 h incubation at 25 °C on Congo Red LB agar *w*/*o* NaCl. rdar-red, dry and rough; bdar—brown, dry and rough; ras—red and smooth; and saw—smooth and white morphotype.

**Figure 2:**
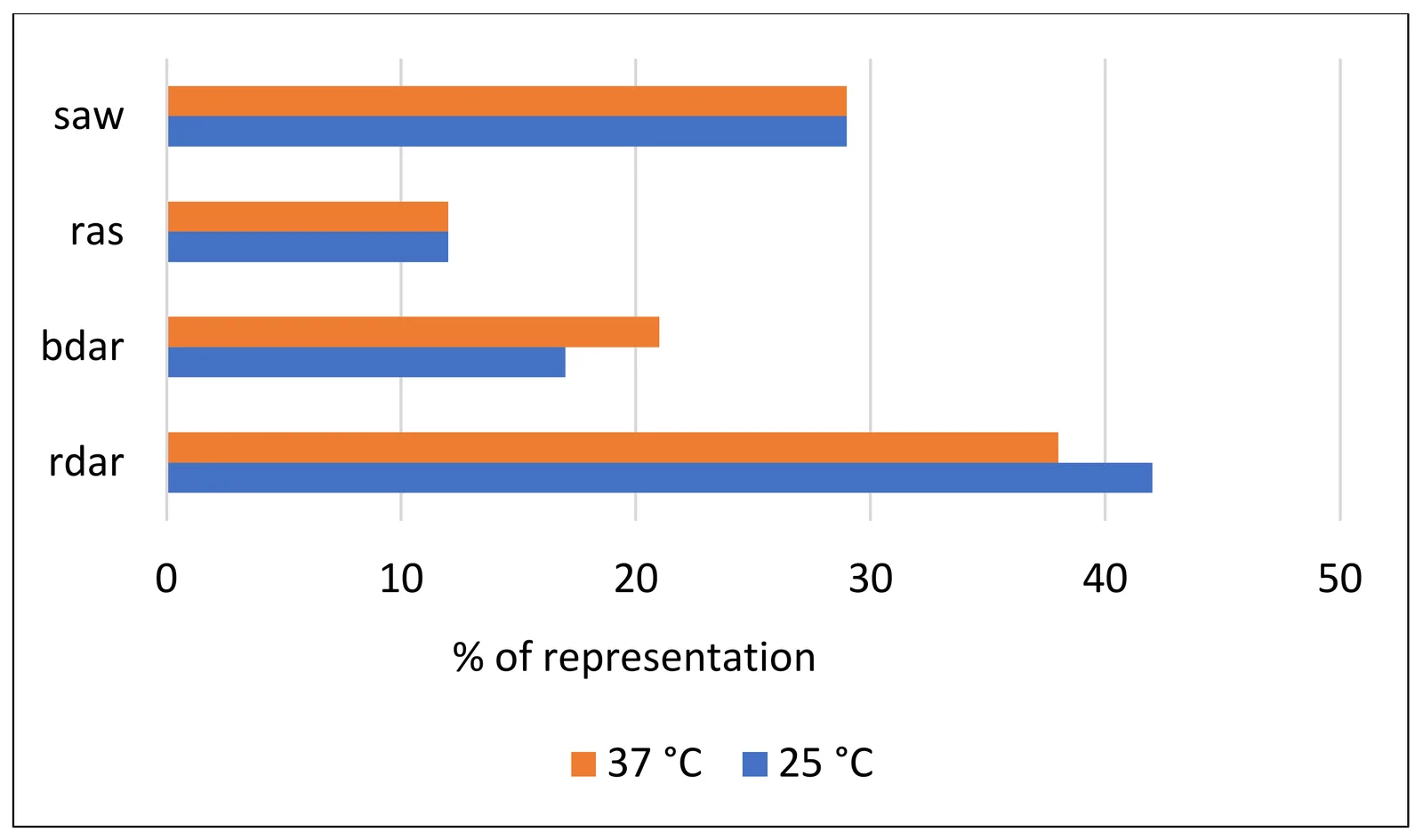
Representation of certain morphotypes of *E. coli* after 96 h incubation at 25 °C and 37 °C on Congo Red LB agar *w*/*o* NaCl.

After 96 h of incubation on Congo Red LB agar without NaCl, four colony morphotypes (rdar, bdar, ras, and saw) were identified among the tested *E. coli* isolates. At 25 °C, the rdar morphotype predominated (10 isolates), followed by saw (7 isolates), bdar (4 isolates), and ras (3 isolates) (Figure 1). A similar distribution was observed at 37 °C, where nine isolates exhibited the rdar morphotype, seven the saw morphotype, five the bdar morphotype, and three the ras morphotype (Figure 2). No pdar or pas morphotypes were detected at either incubation temperature.

Analysis of colony morphotype distributions across thermal conditions revealed significant phenotypic variation within the population. The rdar morphotype predominated overall (42% at 25 °C and 38% at 37 °C), reflecting a high baseline capacity for biofilm-associated matrix production. While ras (12%) and saw (29%) remained invariant across treatments, a temperature-dependent shift occurred between rdar and bdar (which increased from 17% at 25 °C to 21% at 37 °C), demonstrating that thermal cues specifically modulate extracellular matrix architecture.

#### 3.1.2. Crystal Violet Assay

**Figure 3:**
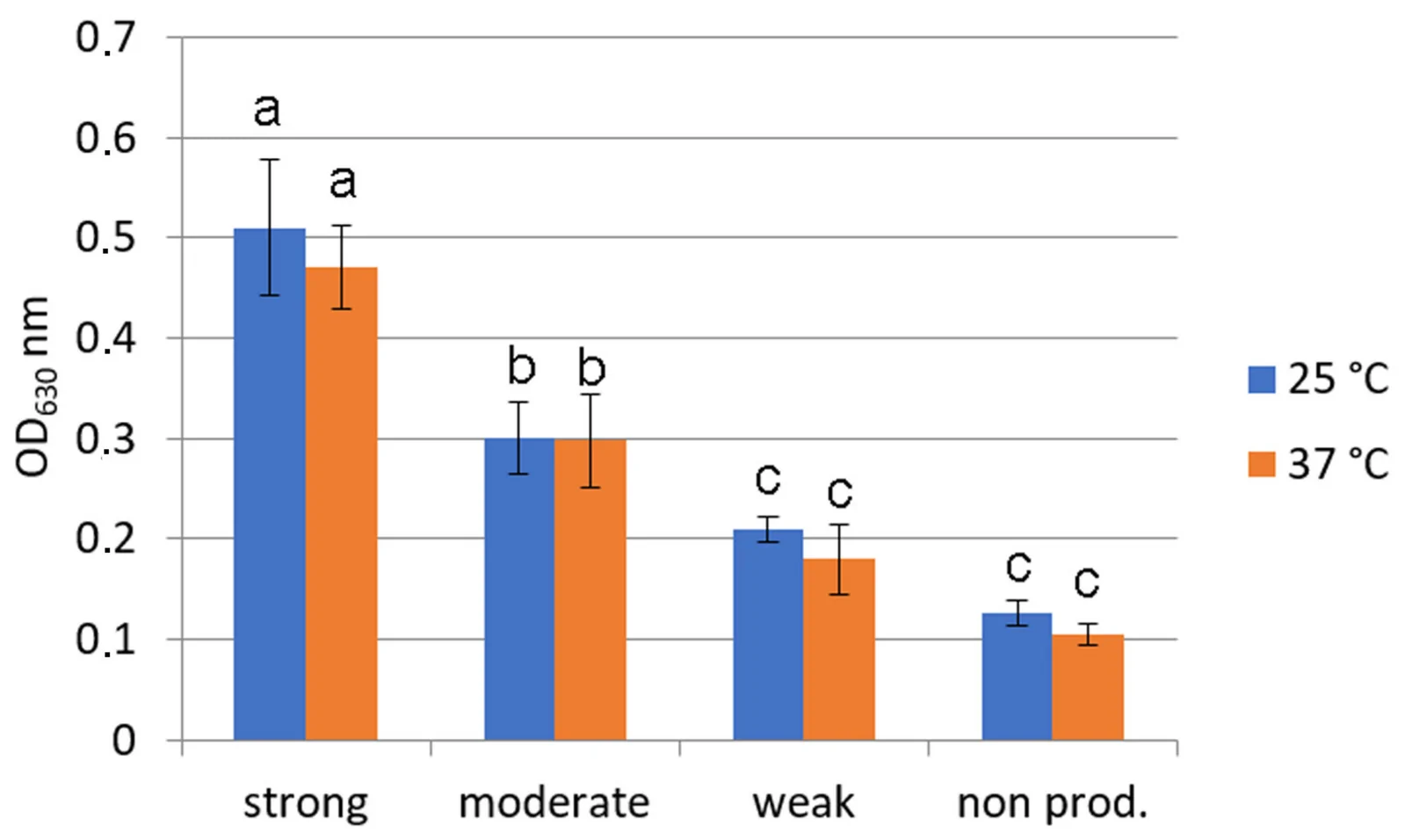
Determined mean absorbance values (OD_630_) with standard deviations (shown on the y axis) in the test on microtiter plates and the representation of *E. coli* isolates according to the strength of biofilm production (shown on the x axis) (incubation in TSB at 25 °C and 37 °C for 48 h).

The biofilm-forming ability of the 24 *E. coli* isolates was evaluated using the crystal violet microtiter plate assay. All isolates were capable of forming biofilms at both incubation temperatures. However, the extent of biofilm formation varied considerably. Based on the ODc threshold values, isolates were classified as strong, moderate, weak, or non-biofilm producers (Figure 3).

Biofilm formation, as evaluated by optical density (OD630) and cut-off values (ODc) of 0.074 at 25 °C and 0.068 at 37 °C, was significantly influenced by incubation temperature (F_1,16_ = 3.88, *p* = 0.066) and phenotypic category (F_3,16_ = 91.82, *p* < 0.0001). At 25 °C, the mean OD_630_ values were 0.510 ± 0.068 ^a^ for strong producers, 0.301 ± 0.036 ^b^ for moderate producers, 0.209 ± 0.013 ^c^ for weak producers, and 0.126 ± 0.012 ^c^ for non-biofilm producers. At 37 °C, slightly lower mean OD_630_ values were recorded at 0.471 ± 0.042 ^a^, 0.298 ± 0.046 ^b^, 0.180 ± 0.035 ^c^, and 0.105 ± 0.010 ^c^ for strong, moderate, weak, and non-biofilm producers, respectively. Tukey’s HSD post hoc test confirmed significant differences among all phenotypic groups (*p* < 0.001) in descending order, with no significant difference only between the weak and non-producing categories (*p* > 0.05), while the temperature-by-category interaction remained non-significant (F_3,16_ = 0.19, *p* > 0.05).

Biofilm formation was influenced by incubation temperature. At 25 °C, 12.5% of isolates were classified as strong biofilm producers, compared with 8.3% at 37 °C. Similarly, the proportion of moderate biofilm producers decreased from 41.7% at 25 °C to 25.0% at 37 °C. In contrast, the percentage of isolates unable to form biofilm increased from 20.8% at 25 °C to 41.7% at 37 °C, while the proportion of weak biofilm producers remained unchanged at 25.0%.

Among the examined isolates, one isolate exhibited the highest biofilm biomass and was therefore selected for all subsequent experiments evaluating the antibacterial and antibiofilm activities of the tested essential oils.

#### 3.1.3. Adhesion of *E. coli* on Lettuce Leaf Surfaces

**Figure 4:**
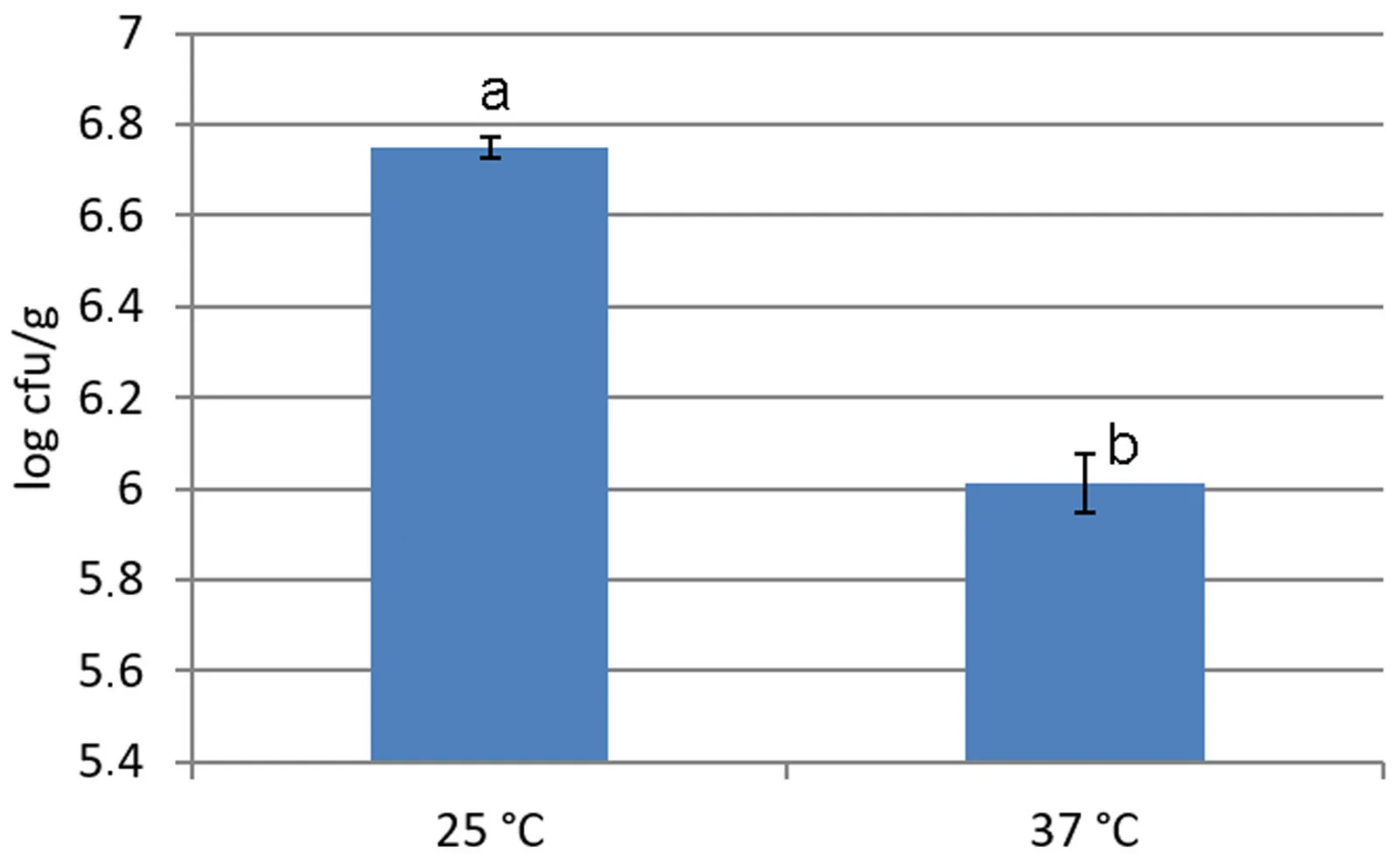
Adherence of *E. coli* to the surface of lettuce leaves at 25 °C and 37 °C after 3 h. Each bar represents the mean ± standard deviation (SD).

The ability of *E. coli* to adhere to lettuce leaf surfaces was evaluated using lettuce leaf coupons prepared as previously described. The results of adherence of *E. coli* to the lettuce leaf coupon surface are shown in Figure 4.

Based on the presented results (Figure 4), the degree of adherence to the surface of lettuce leaves at a temperature of 25 °C and 37 °C was 6.64 log CFU/cm^2^ ± 0.028 and 6.01 log CFU/cm^2^ ± 0.063, respectively. Thus, increasing the incubation temperature from 25 °C to 37 °C reduced bacterial attachment by approximately 10%. Microbial population counts (log cfu/g) were significantly influenced by the incubation temperature (F_1,4_ = 98.26, *p* < 0.001). The highest concentration was observed at 25 °C (6.75 ± 0.03 ^a^), whereas a significant decrease was recorded at 37 °C (6.01 ± 0.07 ^b^), as confirmed by Tukey’s HSD post hoc test (*p* < 0.001).

#### 3.1.4. Scanning Electron Microscopy (SEM) of *E. coli* Biofilms Formed on Lettuce Leaf Surfaces

**Figure 5:**
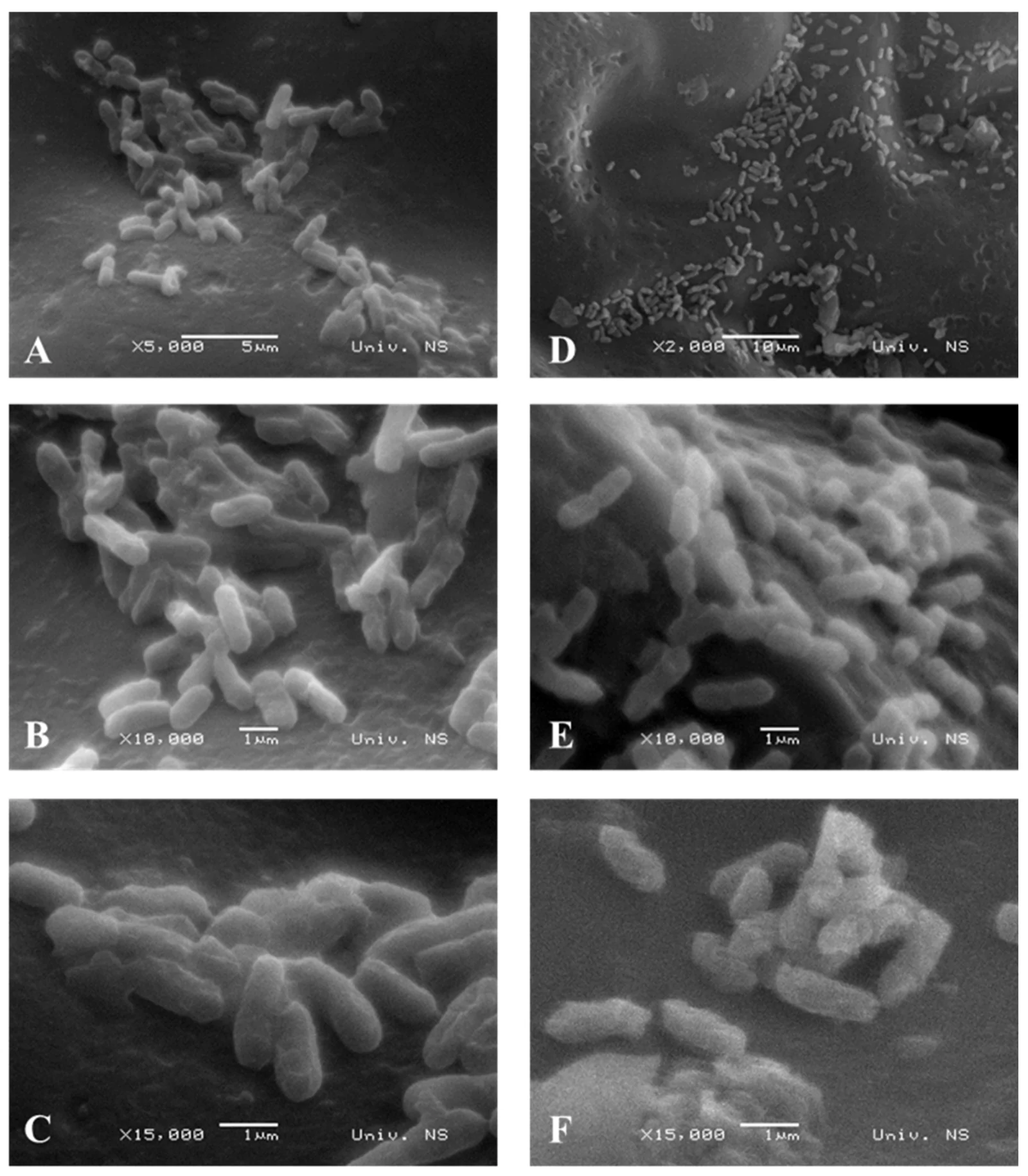
SEM micrographs of *E. coli* biofilm on lettuce leaf coupons at magnifications of 2000×, 5000×, 10,000× and 15,000×: (**A**–**C**) (at 25 °C, 24 h) and (**D**–**F**) (at 37 °C, 24 h).

Representative SEM micrographs of *E. coli* biofilms formed on lettuce leaf surfaces are shown in Figure 5. The SEM micrographs revealed biofilms in the form of an extensive aggregation of bacterial cells on the surfaces of lettuce leaves. Micrographs of lower magnification (Figure 5A,D) show the colonization of the lettuce leaf surface by *E. coli* at both incubation temperatures. Figure 5D, which was taken at the lowest magnification (2000×) and covers a larger area, gives an insight into the uneven adherence of cells on the surface of the leaf. On the micrographs of higher magnification (Figure 5B,E), intense cell aggregation can be observed with the formation of microcolonies partially covered and interconnected by matrix. Comparison of Figure 5B,E revealed a greater amount of extracellular matrix surrounding the microcolonies formed at 25 °C than those formed at 37 °C. Although the biofilm matrix is naturally highly hydrated, it appeared as a compact dried mass in the SEM micrographs (Figure 5C,F). This appearance is an artifact of the SEM sample preparation procedure, which includes successive dehydration steps that remove water from the extracellular matrix.

### 3.2. Chemical Composition of Essential Oils and Their Antibacterial Activity Against E. coli

**Figure 6:**
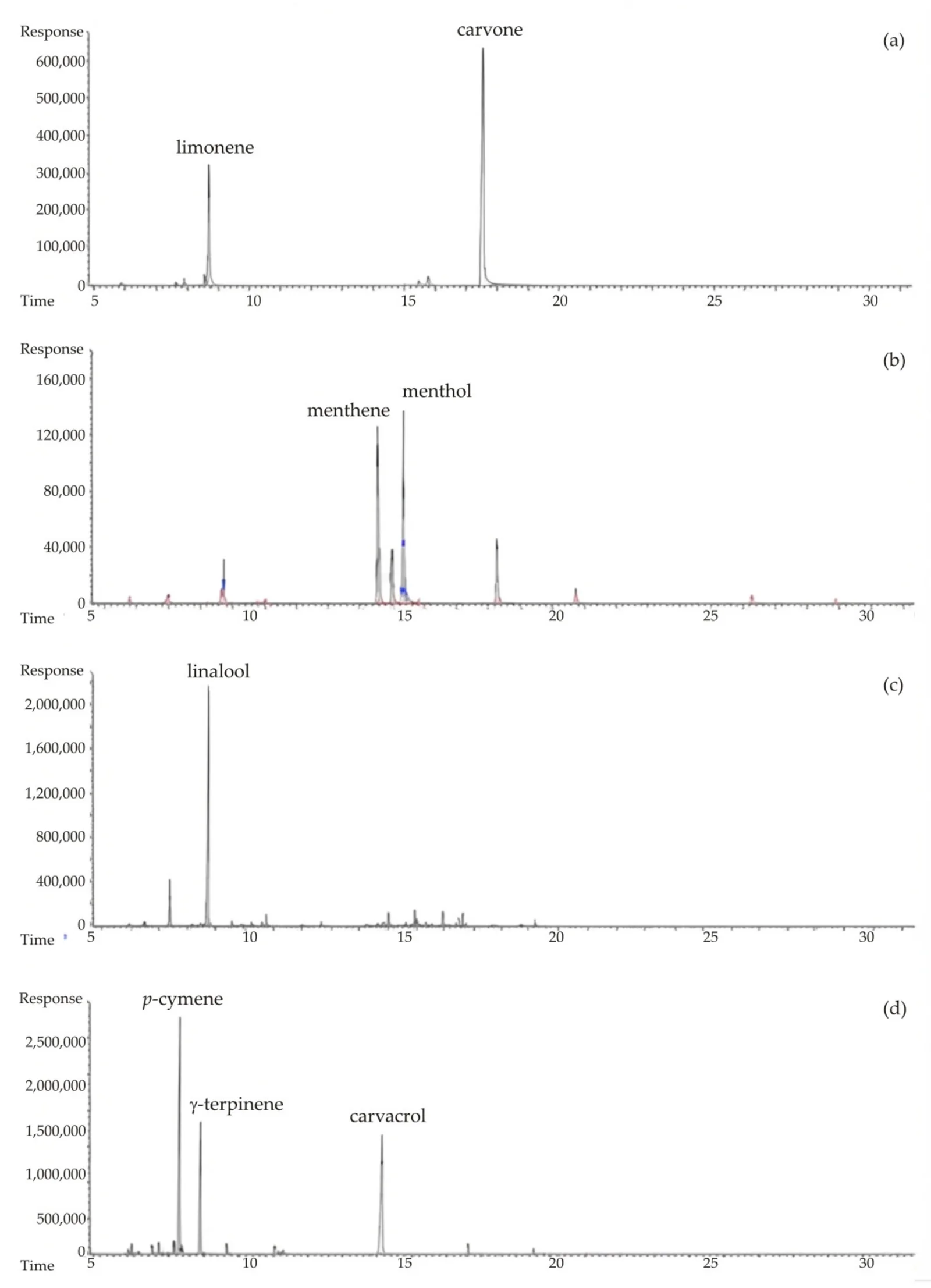
Representative GC–MS chromatograms of the essential oils analyzed in this study: (**a**) dill EO, (**b**) peppermint EO, (**c**) basil EO, and (**d**) winter savory EO.

The chemical composition of the EOs used in this study was previously determined by GC–MS analysis [26]. The major constituents of the dill EO were carvone (66.6%) and limonene (25.3%), and those of peppermint EO were menthol (30.8%) and menthone (28.8%). Linalool was the predominant constituent of basil EO, whereas winter savory was characterized by high proportions of p-cymene (33.8%), carvacrol (32.9%), and γ-terpinene (15.9%). The detailed chemical characterization of these EOs has been reported previously [26], and the major constituents are mentioned here only to provide relevant chemical context for the present study. Representative GC–MS chromatograms of the analysed essential oils are presented in Figure 6.

The antimicrobial activity of the tested essential oils against *E. coli* is presented in Table 1. Among the evaluated essential oils, *A. graveolens* exhibited the highest antibacterial activity, with the lowest MIC and MBC values of 3.55 µL/mL. Essential oils of *O. basilicum* and *S. montana* showed moderate antibacterial activity with MIC values of 7.10 µL/mL, while *M. piperita* demonstrated the lowest antibacterial activity, requiring the highest concentration to inhibit the *E. coli* isolates (14.20 µL/mL). The MIC values determined in this assay were subsequently used to select the concentrations applied in the biofilm experiments, which were performed at 0.5 × MIC, 1 × MIC, and 2 × MIC.

### 3.3. Effect of EOs on Initial Cell Attachment and Preformed Biofilm

#### 3.3.1. Effect of EOs on Initial Cell Attachment In Vitro

**Figure 7:**
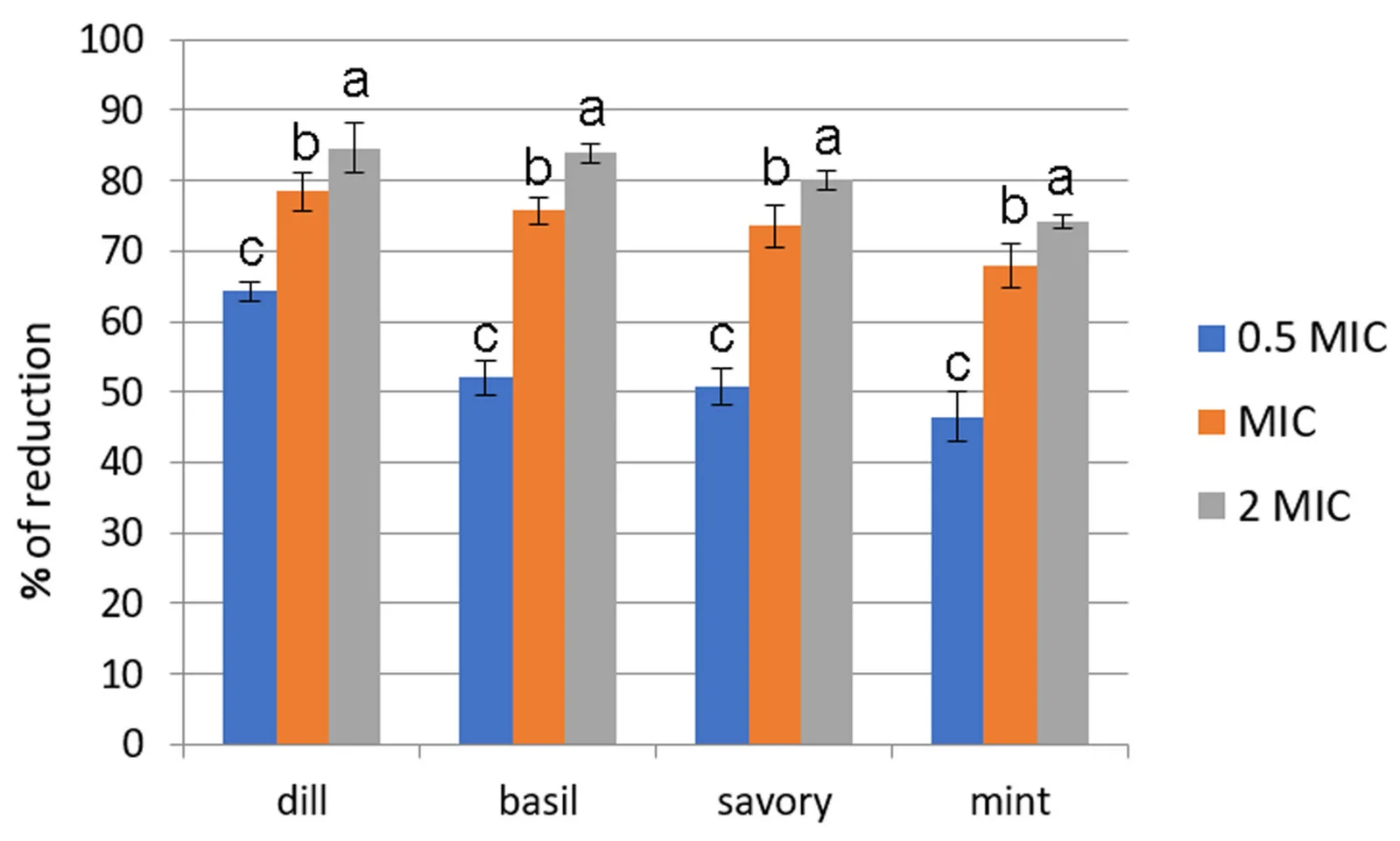
The effect of different concentrations of dill, basil, winter savory and peppermint EOs on initial cell attachment of *E. coli*. Each bar represents the mean ± standard deviation (SD).

In the present study, the EOs of dill, basil, winter savory, and peppermint were evaluated for their antibacterial activity against *E. coli*. The tested EOs exhibited markedly different antibacterial activities against *E. coli*. Dill EO showed the greatest antibacterial activity, with MIC and MBC values of 3.55 µL/mL, whereas peppermint EO was the least effective, exhibiting MIC and MBC values of 14.20 µL/mL and 56.81 μL/mL, respectively. Based on these results, concentrations corresponding to 0.5 × MIC, 1 × MIC, and 2 × MIC were selected to evaluate the effect of the essential oils on the initial attachment of *E. coli* cells (Figure 7). This experimental design enabled the assessment of both sub-inhibitory and inhibitory concentrations, thereby providing insight into how different EO doses interfere with early biofilm establishment.

The tested EOs exhibited a dose-dependent effect on initial *E. coli* adhesion and subsequent biofilm formation, with the overall efficacy at the highest concentration (2 MIC) following the order: dill EO > basil EO > winter savory EO > peppermint EO. At the lowest concentration (0.5 MIC), the reduction in adhered cells ranged from 46.48% to 64.23%, indicating a moderate inhibitory effect even at sublethal levels. Increasing the concentration to MIC resulted in a higher reduction range of 67.87–78.39%, while the highest tested concentration (2 MIC) produced the strongest effect, with reductions between 74.17% and 84.63%.

The percentage of reduction was significantly influenced by both the essential oil type (F_3,24_ = 21.63, *p* < 0.001) and the applied concentration (F_2,24_ = 143.11, *p* < 0.0001). Across all tested botanical extracts, antimicrobial efficacy increased dose-dependently. For the 2 MIC, the highest mean reduction percentages were recorded, reaching 84.5 ± 3.5 ^a^ for dill, 84.0 ± 1.5 ^a^ for basil, 80.0 ± 1.5 ^a^ for winter savory, and 74.2 ± 1.0 ^a^ for peppermint. At the MIC level, reductions decreased significantly to 78.5 ± 2.5 ^b^, 76.0 ± 2.0 ^b^, 73.5 ± 3.0 ^b^, and 68.0 ± 3.0 ^b^, respectively. The lowest reduction rates were observed at 0.5 MIC (64.5 ± 2.0 ^c^ for dill, 52.0 ± 2.5 ^c^ for basil, 50.8 ± 2.5 ^c^ for winter savory, and 46.3 ± 3.5 ^c^ for peppermint). Tukey’s HSD post hoc test confirmed that differences among all three concentration levels were statistically significant (*p* < 0.05), while the interaction between essential oil type and concentration was non-significant (F_6,24_ = 1.24, *p* > 0.05).

#### 3.3.2. Effect of EOs on Preformed Biofilm In Vitro

**Figure 8:**
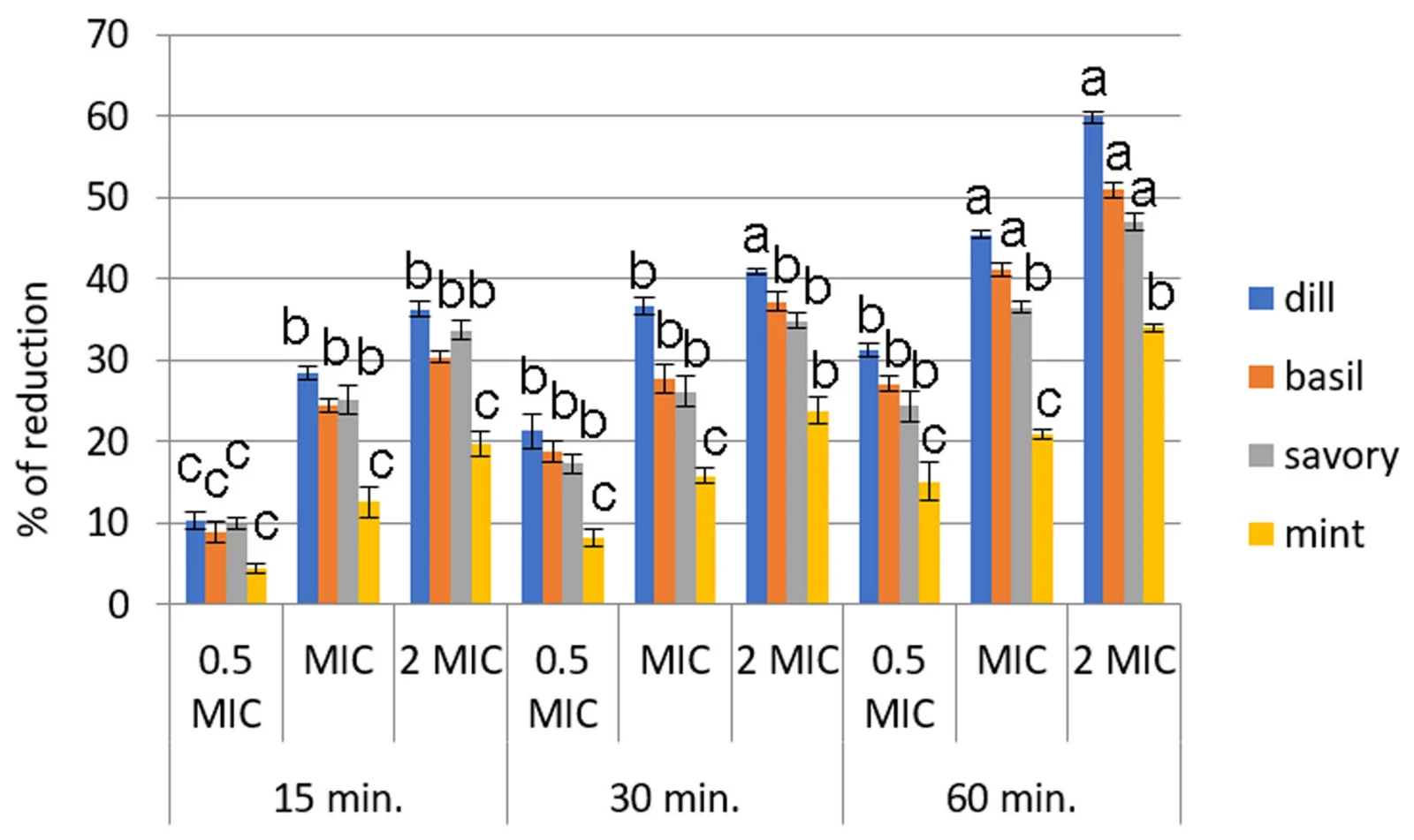
The influence of different concentrations of dill, basil, winter savory and peppermint EO on the previously formed *E. coli* biofilm as a function of time. Each bar represents the mean ± standard deviation (SD).

The effects of the tested EOs on preformed 48 h *E. coli* biofilms are presented in Figure 8. The results clearly demonstrate that biofilm eradication was both concentration- and time-dependent, with greater reductions observed at higher EO concentrations and longer exposure times. The highest antibiofilm activity was achieved at 2 MIC after 60 min of treatment, indicating that effective disruption of established biofilms requires both sufficient EO concentration and prolonged contact time.

To facilitate comparison among treatments, the minimum biofilm eradication concentration (MBEC_50_), defined as the lowest concentration capable of reducing the total biofilm biomass by at least 50%, was determined. For the tested *E. coli* strain, MBEC_50_ was achieved only with dill and basil EOs at 2 MIC following 60 min of exposure. In contrast, winter savory and peppermint EOs failed to reach the MBEC_50_ threshold under any of the tested conditions, highlighting their comparatively weaker antibiofilm activity.

Among all treatments, dill EO exhibited the strongest effect, reducing biofilm biomass by 59.90% after 60 min at 2 MIC. Under the same conditions, basil EO achieved a 50.86% reduction, whereas winter savory and peppermint EOs reduced biofilm biomass by 47.03% and 34.00%, respectively.

As expected, the weakest antibiofilm effects were observed at the lowest EO concentration (0.5 MIC) and shortest exposure time (15 min). Under these conditions, reductions in biofilm biomass were limited to 10.30%, 8.93%, 9.86%, and 4.33% for dill, basil, winter savory, and peppermint EOs, respectively.

At MIC and 30 min exposure, biofilm reduction reached 36.67%, 27.70%, 26.13%, and 15.76% for dill, basil, winter savory, and peppermint EOs, respectively. Although longer exposure generally enhanced antibiofilm activity, the increase was not proportional to treatment duration. For example, extending the exposure from 30 to 60 min did not result in a twofold increase in biofilm reduction, suggesting that EO activity reaches a plateau after an initial period of rapid action.

The percentage reduction of microorganisms was significantly affected by contact time (F_2,72_ = 458.28, *p* < 0.0001), essential oil concentration (F_2,72_ = 775.75, *p* < 0.0001), and plant extract type (F_3,72_ = 259.10, *p* < 0.0001). At 15 min, the mean reduction values for dill, basil, winter savory, and peppermint were 10.0 ± 1.2 ^c^, 9.0 ± 1.0 ^c^, 10.0 ± 0.8 ^c^, and 4.5 ± 0.5 ^c^ at 0.5 MIC; 28.5 ± 1.0 ^b^, 24.5 ± 1.2 ^b^, 25.0 ± 1.8 ^b^, and 12.5 ± 2.0 ^c^ at MIC; and 36.0 ± 1.1 ^b^, 30.5 ± 1.5 ^b^, 33.5 ± 1.4 ^b^, and 19.5 ± 1.6 ^c^ at 2 MIC, respectively. At 30 min, reductions were recorded at 21.2 ± 1.9 ^b^, 18.5 ± 1.4 ^b^, 17.5 ± 1.1 ^b^, and 8.2 ± 1.3 ^c^ (0.5 MIC); 36.5 ± 1.2 ^b^, 27.5 ± 1.9 ^b^, 26.0 ± 2.0 ^b^, and 15.8 ± 1.2 ^c^ (MIC); and 41.0 ± 0.5 ^a^, 37.0 ± 1.4 ^b^, 34.5 ± 1.2 ^b^, and 23.5 ± 1.9 ^b^ (2 MIC). At 60 min, the highest efficacy was observed, with mean reductions reaching 31.0 ± 1.0 ^b^, 27.0 ± 1.1 ^b^, 24.5 ± 1.8 ^b^, and 15.0 ± 2.2 ^c^ (0.5 MIC); 45.5 ± 0.6 ^a^, 41.0 ± 1.0 ^a^, 36.5 ± 1.0 ^b^, and 20.8 ± 0.8 ^c^ (MIC); and 60.0 ± 0.7 ^a^, 51.0 ± 1.2 ^a^, 47.0 ± 1.1 ^a^, and 34.0 ± 0.5 ^b^ (2 MIC). Tukey’s HSD post hoc test confirmed that antimicrobial reduction increased significantly with prolonged contact time (60 min > 30 min > 15 min) and higher concentrations (2 MIC > MIC > 0.5 MIC), while dill and winter savory exhibited the highest overall extract efficacy (*p* < 0.01).

#### 3.3.3. Efficacy of Essential Oils Against Preformed Biofilms on Lettuce Leaves

**Figure 9:**
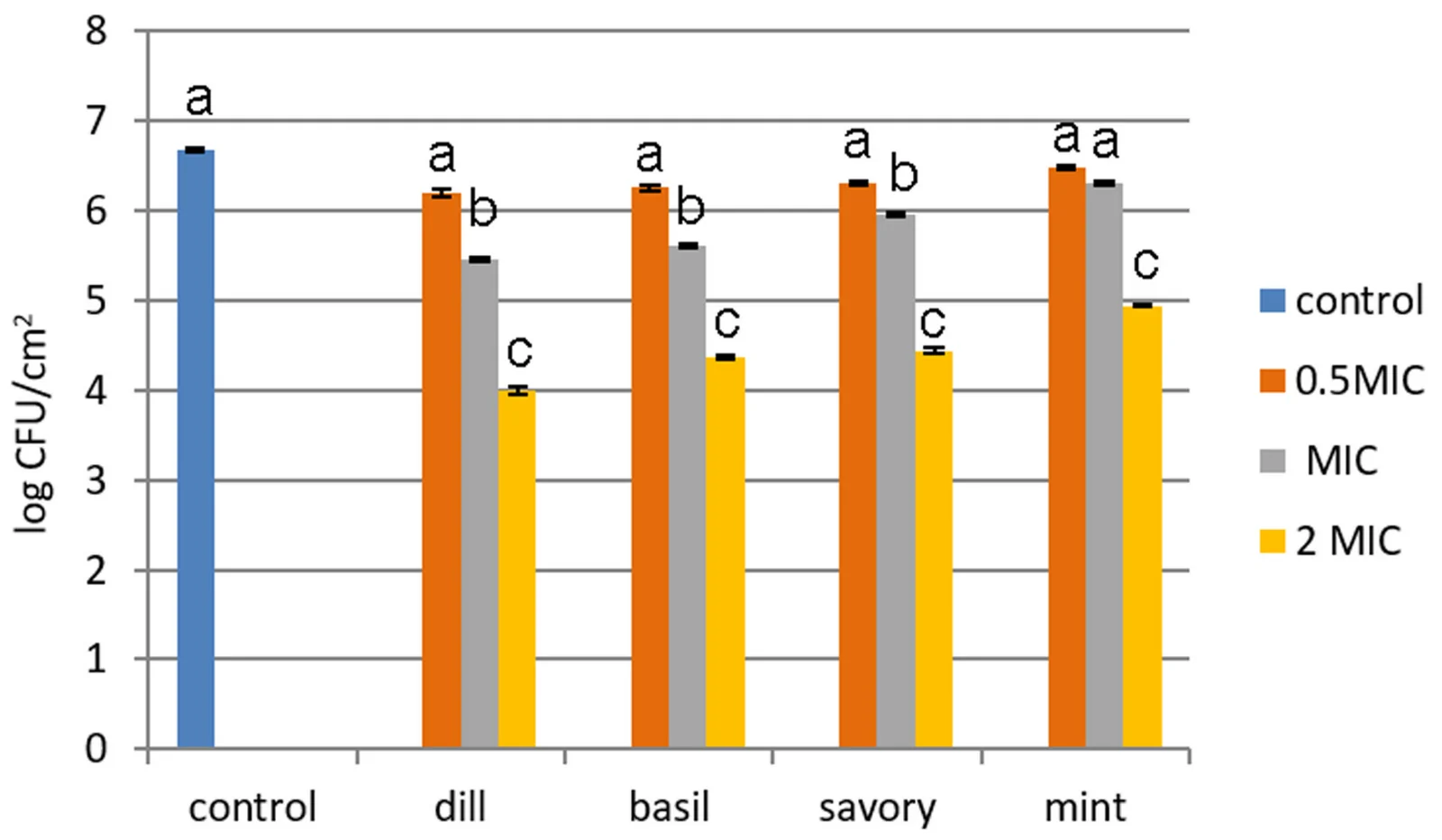
Biofilm cells grown at 25 °C for 24 h on lettuce leaf surface before and after EOs treatment (0.5 MIC, MIC, 2MIC level) for 1 h at room temperature. The control represents average bacterial counts in biofilm before treatment with EOs.

The effect of EOs on biofilm *E. coli* on the lettuce leaf surface was analyzed from the aspect of determining the number of viable cells after exposure of the previously formed biofilm to concentrations of EO of 0.5 MIC, MIC and 2 MIC (Figure 9).

Microbial surface populations (log CFU/cm^2^) were significantly reduced by essential oil treatments (F_4,32_ = 46.37, *p* < 0.0001) and concentration levels (F_3,32_ = 606.74, *p* < 0.0001). The untreated control exhibited the highest recovery count at 6.68 ± 0.03 ^a^. For dill, microbial counts were 6.18 ± 0.05 ^a^ (0.5 MIC), 5.45 ± 0.04 ^b^ (MIC), and 4.00 ± 0.05 ^c^ (2 MIC). For basil, counts were recorded at 6.25 ± 0.04 ^a^, 5.62 ± 0.03 ^b^, and 4.38 ± 0.03 ^c^, respectively. For winter savory, values reached 6.30 ± 0.03 ^a^, 5.98 ± 0.04 ^b^, and 4.45 ± 0.04 ^c^, while peppermint treatments yielded 6.48 ± 0.03 ^a^, 6.31 ± 0.02 ^a^, and 4.96 ± 0.03 ^c^ across corresponding concentrations. Tukey’s HSD post hoc test confirmed that microbial survival decreased significantly with increasing extract concentration (2 MIC < MIC < 0.5 MIC, *p* < 0.01).

**Figure 10:**
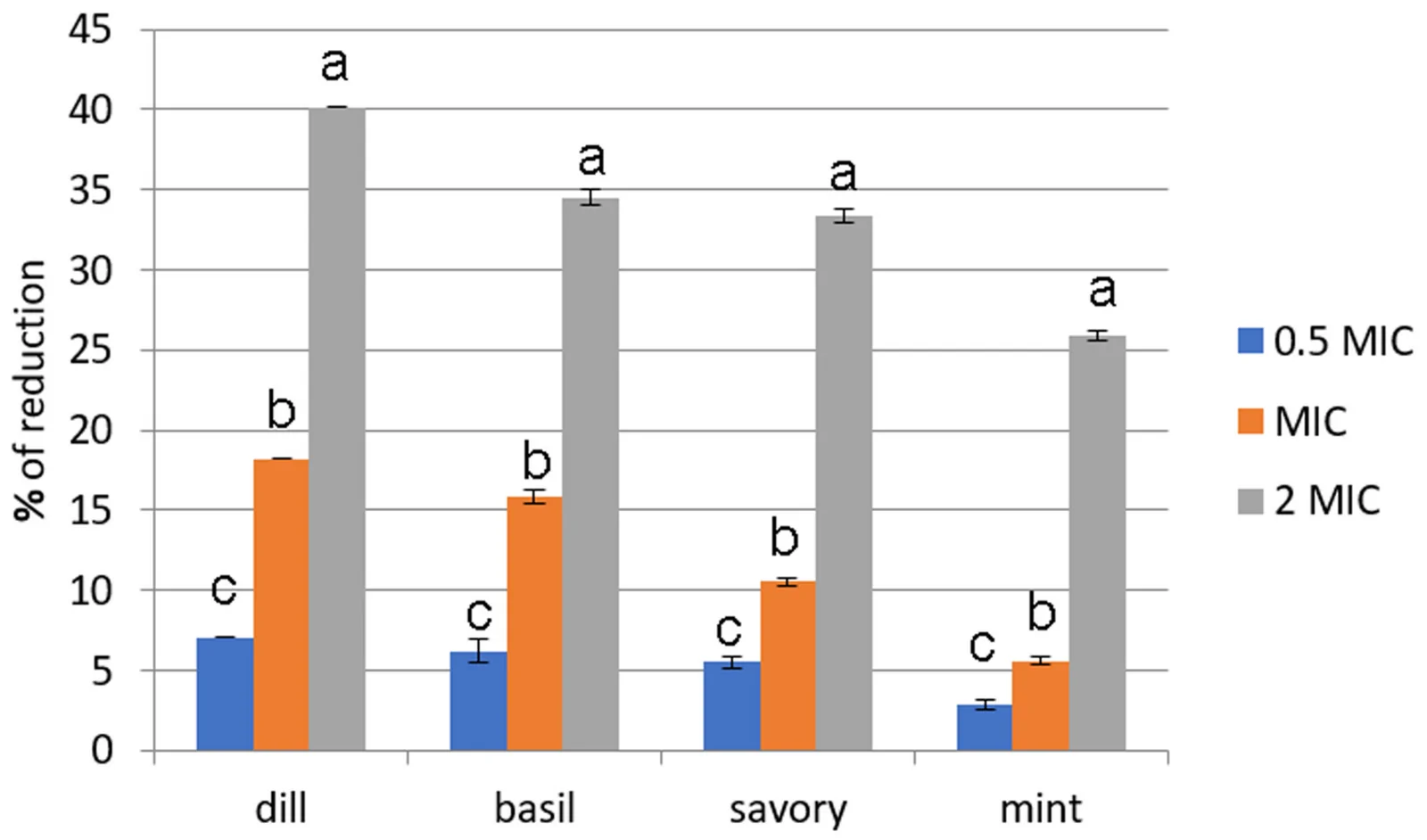
The effect of different concentrations of dill, basil, winter savory and peppermint EOs on preformed *E. coli* biofilm on lettuce leaf surface. Each bar represents the mean ± standard deviation (SD).

**Figure 11:**
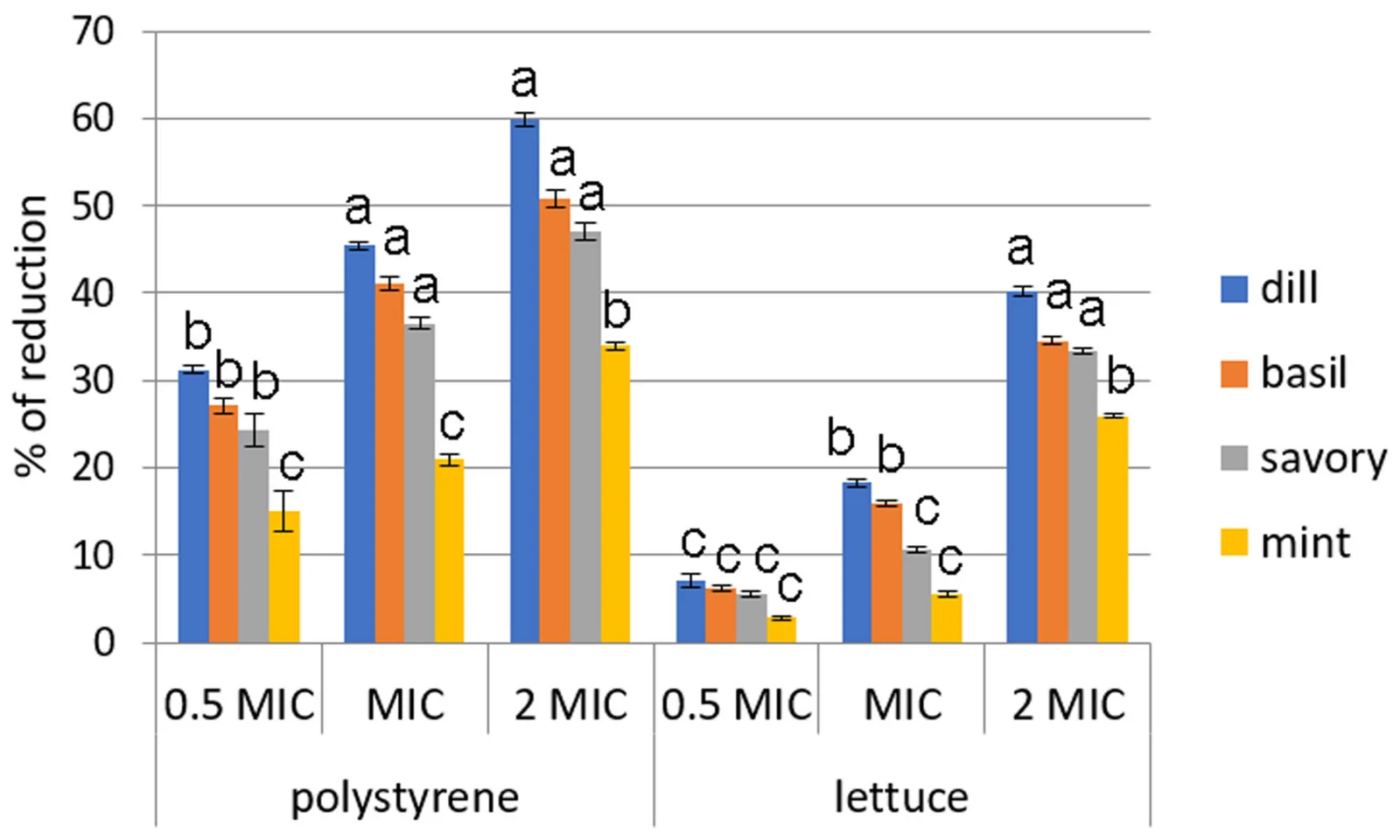
Comparative analysis of the reduction in *E. coli* biofilm formed in a polystyrene microtiter plate and on the lettuce leaf surface.

The effects of EO treatments on viable biofilm cells formed on lettuce leaves are presented in Figure 10. Comparison with biofilms formed on polystyrene microtiter plates demonstrated lower reductions on lettuce leaf surfaces (Figure 11).

The percentage reduction of microorganisms was significantly influenced by the essential oil type (F_3,24_ = 1465.38, *p* < 0.0001), concentration (F_2,24_ = 22,124.24, *p* < 0.0001), and their interaction (F_6,24_ = 149.63, *p* < 0.0001). For dill, reduction rates were 7.0 ± 0.1 ^c^ (0.5 MIC), 18.2 ± 0.1 ^b^ (MIC), and 40.0 ± 0.1 ^a^ (2 MIC). For basil, values reached 6.2 ± 0.8 ^c^, 15.8 ± 0.5 ^b^, and 34.5 ± 0.6 ^a^, respectively. Winter savory treatments recorded reductions of 5.6 ± 0.3 ^c^, 10.5 ± 0.3 ^b^, and 33.4 ± 0.4 ^a^, while peppermint yielded the lowest percentages at 2.9 ± 0.3 ^c^, 5.6 ± 0.3 ^b^, and 26.0 ± 0.3 ^a^. Tukey’s HSD post hoc test confirmed that reduction efficacy increased significantly across all concentration levels (2 MIC > MIC > 0.5 MIC, *p* < 0.001).

The percentage reduction of microorganisms was significantly influenced by surface type (F_1,48_ = 10,671.13, *p* < 0.0001), essential oil concentration (F_2,48_ = 6739.09, *p* < 0.0001), and plant extract type (F_3,48_ = 1362.81, *p* < 0.0001). On polystyrene surfaces, mean reduction rates at 0.5 MIC reached 31.0 ± 0.8 ^b^ (dill), 27.2 ± 0.9 ^b^ (basil), 24.2 ± 1.9 ^b^ (winter savory), and 15.0 ± 2.2 ^c^ (peppermint); at MIC, values were 45.5 ± 0.4 ^a^ (dill), 41.0 ± 1.0 ^a^ (basil), 36.2 ± 1.0 ^a^ (winter savory), and 21.0 ± 0.5 ^c^ (peppermint); and at 2MIC, they reached 60.0 ± 0.6 ^a^ (dill), 51.0 ± 0.9 ^a^ (basil), 47.0 ± 1.1 ^a^ (winter savory), and 34.0 ± 0.4 ^b^ (peppermint). On lettuce surfaces, reductions were lower, recording 7.0 ± 0.8 ^c^ (dill), 6.2 ± 0.4 ^c^ (basil), 5.6 ± 0.4 ^c^ (winter savory), and 2.8 ± 0.2 ^c^ (peppermint) at 0.5 MIC; 18.2 ± 0.4 ^b^ (dill), 15.8 ± 0.4 ^b^ (basil), 10.5 ± 0.4 ^c^ (winter savory), and 5.6 ± 0.4 ^c^ (peppermint) at MIC; and 40.0 ± 0.6 ^a^ (dill), 34.5 ± 0.6 ^a^ (basil), 33.4 ± 0.4 ^a^ (winter savory), and 26.0 ± 0.4 ^b^ (peppermint) at 2 MIC. Tukey’s HSD post hoc test confirmed that antimicrobial efficiency was significantly greater on polystyrene than on lettuce, and increased progressively with higher essential oil concentrations, *p* < 0.01.

#### 3.3.4. SEM of *E. coli* Biofilm Formed on the Surface of Lettuce Leaves After Exposure of EOs

**Figure 12:**
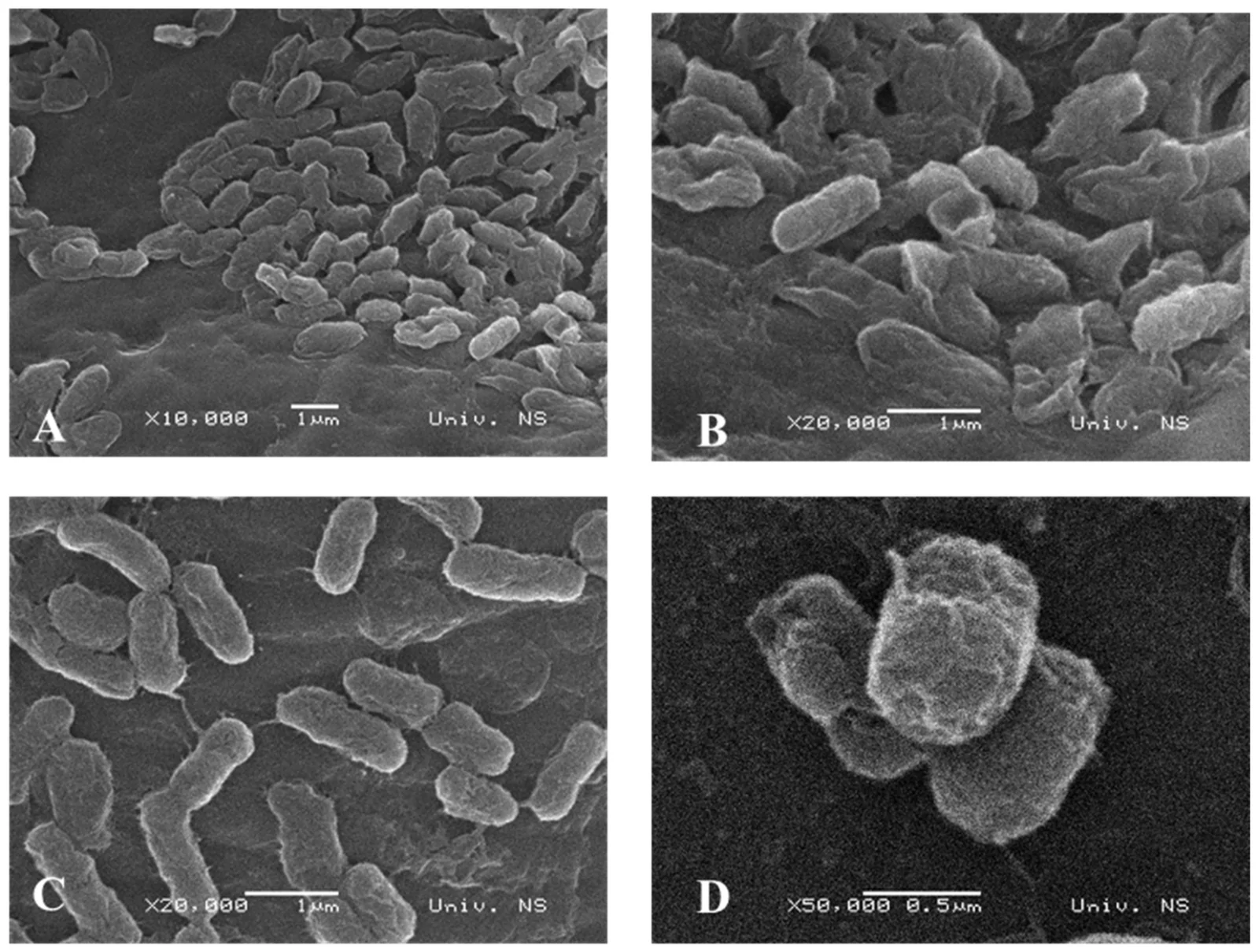
SEM micrographs of *E. coli* biofilm after exposure to dill EO at a concentration of 2 MIC ((**A**) magnification 10,000×, (**B**,**C**) 20,000×, (**D**) 50,000×).

Representative SEM micrographs (Figure 12) revealed marked structural alterations in *E. coli* biofilms following treatment with the tested EOs. Figure 12A,C show fragmented biofilm structures consisting of smaller microcolonies with a marked reduction in large bacterial aggregates compared with the untreated control. In Figure 12B shows markedly deformed cells of *E. coli* due to the leakage of intracellular contents under the influence of EO [34]. Figure 12D further demonstrates severe damage to the bacterial envelope, manifested as a rough and irregular cell surface. The structural damage observed in the SEM micrographs is consistent with the well-established mechanism of action of EOs. The SEM observations corroborate the results obtained by viable cell enumeration, demonstrating that treatment with EOs not only reduced the number of viable biofilm-associated cells but also caused extensive structural damage to the remaining biofilm. The disruption of microcolonies and deformation of bacterial cells provide visual confirmation of the antibiofilm activity observed in the quantitative assays.

### 3.4. Statistical Analysis of the Results

**Figure 13:**
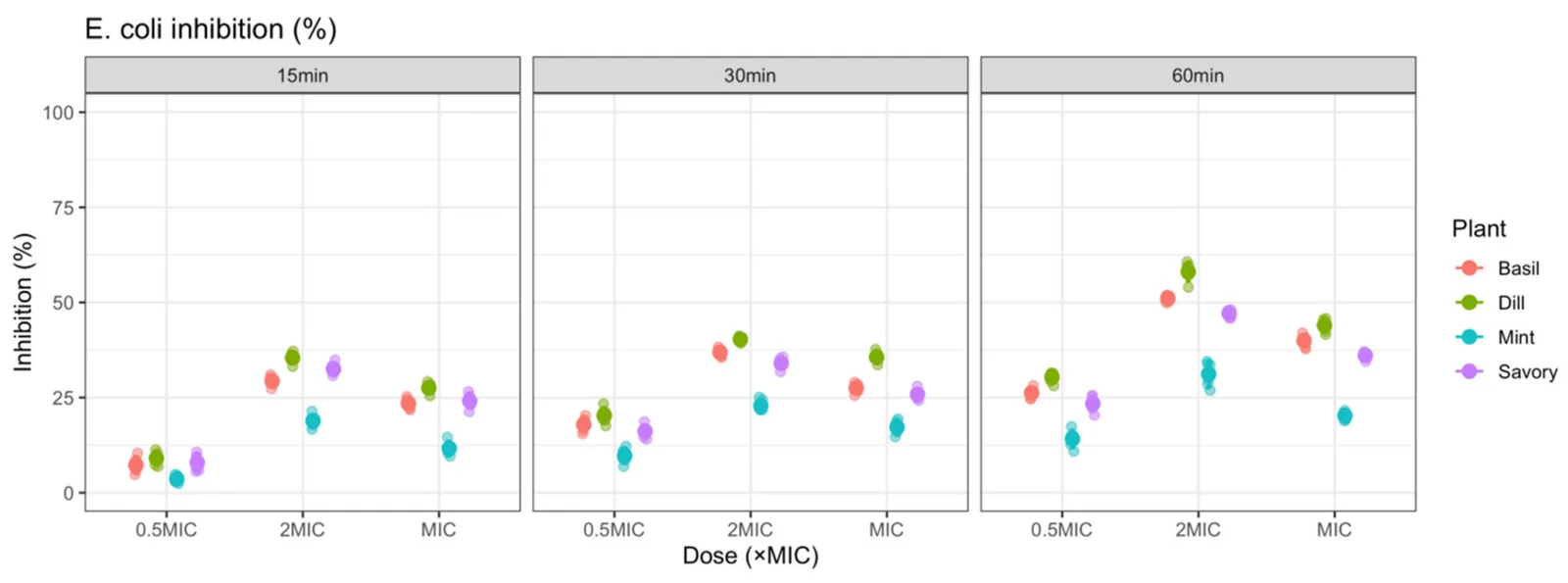
Mean inhibition of *E. coli* (%) as affected by EOs and dose across different exposure times. Points represent mean values, while error bars indicate 95% confidence intervals. The figure demonstrates dose-dependent inhibition patterns and plant-specific response behaviour.

The experimental dataset exhibited a fully balanced factorial structure, with equal replication across all Plant × Dose × Time combinations. Descriptive statistics revealed a consistent increase in *E. coli* inhibition with increasing dose and exposure time for all tested EOs. The variability within treatment groups was relatively low, as indicated by small standard deviations and narrow confidence intervals, suggesting good repeatability of the measurements (Figure 13).

**Figure 14:**
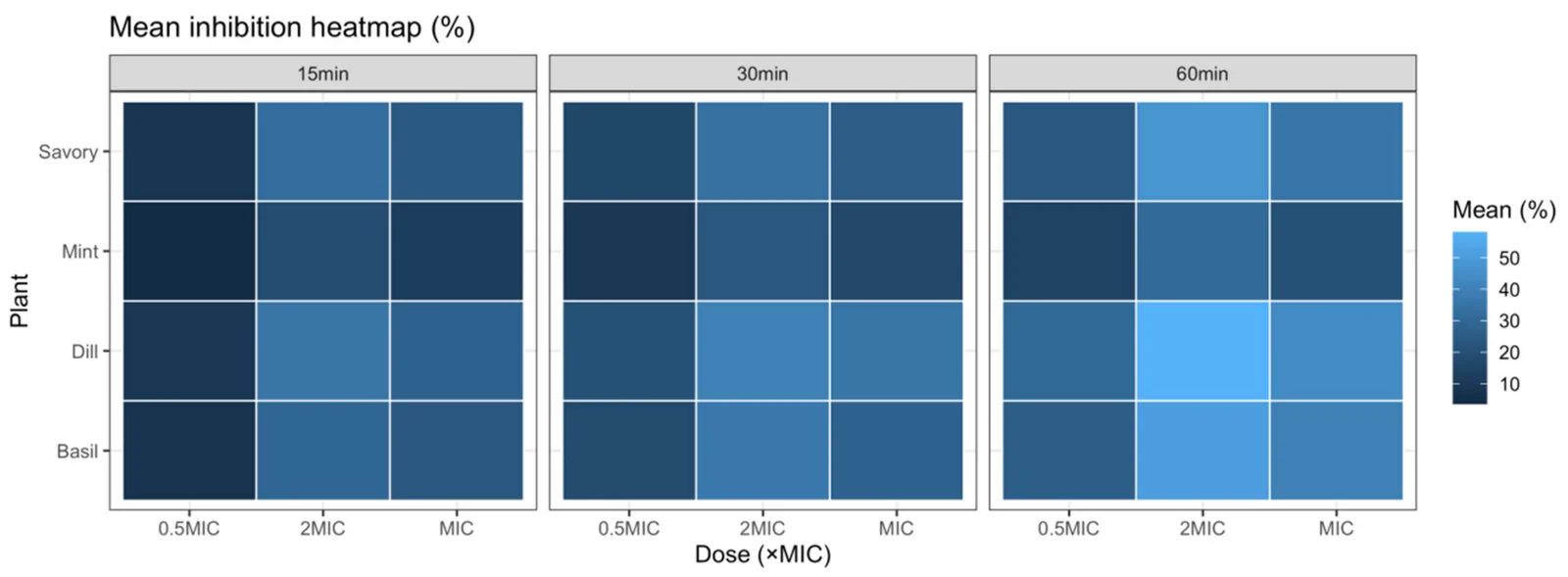
Heatmap representation of mean *E. coli* inhibition (%) for each EO × Dose × Time combination. Colour intensity corresponds to inhibition magnitude, illustrating the combined influence of plant extract, concentration, and exposure time on antimicrobial activity.

Three-way analysis of variance (ANOVA) demonstrated highly significant main effects of EO, dose, and exposure time (*p* < 0.001). Among the evaluated factors, dose exerted the strongest statistical influence on inhibition, followed by exposure time and EO. This finding indicates that concentration was the dominant determinant of antimicrobial efficacy, which is consistent with classical dose–response behaviour observed for plant-derived antimicrobial compounds. In addition to the main effects, significant two-way interactions (Plant × Dose, Plant × Time, Dose × Time) were observed, confirming that the inhibitory response depended on the combined influence of experimental factors. The presence of a statistically significant three-way interaction further indicates that the magnitude of inhibition varied according to specific EO × Dose × Time combinations (Figure 14). These interaction effects demonstrate that antimicrobial performance cannot be adequately explained by individual factors alone but rather by their combined action. Levene’s test indicated homogeneity of variances (*p* > 0.05), supporting the validity of the ANOVA model. Residual diagnostics showed no systematic deviations from model assumptions, confirming the adequacy of the statistical approach. Tukey’s post hoc comparisons revealed statistically significant differences among plant EOs within individual Dose × Time conditions. Differences between doses were consistently significant, confirming a strong concentration-dependent response. Exposure time also significantly influenced inhibition, although the magnitude of this effect varied among EOs, suggesting plant-specific response patterns. The observed increase in inhibition with both dose and exposure time aligns with well-established antimicrobial mechanisms of plant-derived bioactive compounds, where higher concentrations and longer contact times enhance bacterial growth suppression. However, the differences detected among EOs indicate that antimicrobial efficacy is also governed by plant-specific chemical composition and bioactive profiles. Overall, the results confirm that antimicrobial activity against *E. coli* is strongly influenced by the combined effects of plant type, dose, and exposure time. These findings highlight the importance of multi-factor experimental designs when evaluating antimicrobial efficacy and support the hypothesis that optimal inhibitory performance arises from the interaction of concentration, treatment duration, and botanical source.

## 4. Discussion

The persistence of *E. coli* on fresh produce remains a major food safety challenge because bacterial attachment and biofilm formation substantially increase survival on plant surfaces and reduce the effectiveness of conventional washing and sanitation procedures. Although the antimicrobial activity of essential oils against planktonic foodborne pathogens has been extensively investigated [16,26,35,36], their ability to interfere with *E. coli* biofilm development on fresh produce surfaces remains comparatively less understood. A major challenge in evaluating natural antibiofilm agents is that biofilm behavior is strongly influenced by bacterial phenotype, environmental conditions, and the characteristics of the colonized surface. The present study addressed this knowledge gap by integrating phenotypic characterization of biofilm formation, quantitative assessment of bacterial attachment to lettuce leaves, microscopic visualization of biofilm architecture, and evaluation of essential oils against both initial attachment and established biofilms. The findings demonstrate that environmental temperature strongly influences *E. coli* persistence on lettuce surfaces and that essential oils, particularly dill essential oil, exhibit greater potential as preventive antibiofilm agents.

The predominance of the rdar morphotype among the examined *E. coli* isolates represents an important finding because this phenotype is directly associated with the production of the major extracellular matrix components required for stable biofilm development, whereas bdar, ras, and saw colonies displayed the phenotypic characteristics previously described by Römling et al., 2005 [24]. The rdar phenotype reflects simultaneous expression of curli fimbriae and cellulose, which contribute to irreversible attachment, cell aggregation, and structural stability of the biofilm matrix [23,37]. The high frequency of this morphotype among isolates originating from food-related sources indicates that environmental and animal-associated *E. coli* populations may possess considerable capacity for surface persistence. Similar predominance of the rdar phenotype has previously been reported among *E. coli* isolates from bovine mastitis, suggesting that this phenotype represents a widespread adaptive strategy among diverse *E. coli* populations [38].

Interestingly, the majority of isolates retained the rdar phenotype at both 25 °C and 37 °C, indicating that extracellular matrix production was not completely restricted to environmental temperatures. Although curli and cellulose production in *E. coli* is commonly associated with temperatures below 30 °C, regulation of matrix synthesis is highly dependent on strain background and environmental signals. The activity of the transcriptional regulator CsgD, which coordinates curli and cellulose biosynthesis, is controlled by a complex regulatory network responding to temperature, nutrient availability, osmolarity, and stress conditions [24,39,40]. Therefore, the persistence of the rdar phenotype at 37 °C observed in the present study suggests that the examined isolates possess regulatory flexibility that allows maintenance of biofilm-associated traits under different ecological conditions.

The crystal violet assay confirmed that temperature significantly influenced the extent of biofilm development. Although all isolates were capable of producing biofilms at both incubation temperatures, greater biomass accumulation was observed at 25 °C. This finding indicates that environmental conditions favourable for extracellular matrix synthesis may promote a sessile lifestyle, even when bacterial growth itself is not optimal. In contrast, incubation at 37 °C, which represents the optimal growth temperature for *E. coli*, resulted in a higher proportion of weak or non-biofilm-producing isolates. This suggests that biofilm formation is not simply a consequence of increased bacterial growth but rather an adaptive response regulated by environmental conditions. Under nutrient-rich conditions, bacterial cells may preferentially allocate metabolic resources toward rapid multiplication, whereas moderate environmental stress may stimulate investment in extracellular matrix production as a survival strategy [41]. The greater bacterial attachment observed on lettuce leaves at 25 °C further supports this interpretation. The enhanced adhesion was consistent with the predominance of biofilm-associated phenotypes and the greater biofilm biomass detected at the lower temperature. These findings suggest that environmental conditions favouring extracellular matrix production may also promote bacterial attachment to plant surfaces.

The enhanced biofilm formation observed at 25 °C was also reflected in the lettuce attachment assay, where bacterial adhesion was significantly greater than at 37 °C. Attachment to plant surfaces represents the initial critical step in colonization, during which bacterial cells transition from reversible interactions to stable surface association. This process involves a coordinated contribution of flagella, fimbriae, curli fibers, cellulose, and other extracellular matrix components [42,43]. Therefore, the greater adhesion observed at 25 °C is consistent with the higher prevalence of matrix-producing phenotypes and increased biofilm biomass detected under the same conditions. From a food safety perspective, these findings are particularly relevant because fresh produce is frequently exposed to temperatures considerably below the mammalian host temperature during harvesting, processing, transportation, and retail handling. Such conditions may unintentionally promote extracellular matrix production and facilitate bacterial persistence on vegetable surfaces. Once established, biofilms can protect bacterial populations from environmental fluctuations and significantly reduce the effectiveness of subsequent decontamination treatments [43,44].

The SEM observations provided further evidence that temperature-dependent differences in biofilm formation were associated with changes in biofilm architecture. Biofilms developed at 25 °C exhibited more extensive bacterial aggregation and a more pronounced extracellular matrix compared with those formed at 37 °C, supporting the quantitative findings obtained by the crystal violet assay and lettuce attachment experiments. Although SEM preparation involves dehydration steps that inevitably alter the native hydrated structure of the extracellular polymeric substance (EPS), the observed differences between incubation conditions clearly demonstrate that environmental temperature affects the structural organization and maturation of *E. coli* biofilms. The heterogeneous distribution of bacterial cells across the lettuce surface further confirms the complexity of plant-associated biofilms. Bacterial cells preferentially colonize protected surface regions, including epidermal junctions, grooves, and irregularities, where they are less exposed to mechanical removal and environmental stresses [45].

A major objective of this study was to evaluate whether essential oils could provide an effective natural strategy for controlling *E. coli* biofilms under conditions relevant to fresh produce. The tested essential oils differed considerably in their antibacterial activity, with dill essential oil exhibiting the highest inhibitory effect, followed by basil, winter savory, and peppermint essential oils.

This ranking was also observed in the treatment of preformed biofilms. At 2 × MIC after 60 min of exposure, dill EO produced the greatest reduction in biofilm biomass (59.90%), followed by basil EO (50.86%), savory EO (47.03%), and peppermint EO (34.00%), indicating that the chemical characteristics responsible for inhibition of planktonic cells also influence the ability of essential oils to interfere with biofilm development and persistence [26]. In particular, the high activity of dill EO may be partly related to its high proportion of carvone and limonene, while the antimicrobial activity of winter savory EO may be associated with its substantial carvacrol content. Similarly, linalool in basil EO and menthol and menthone in peppermint EO may contribute to their observed antibacterial effects. For example, dill oil is exceptionally rich in D-carvone, a volatile monoterpene ketone whose geometric configuration confers higher reactivity against microbial enzyme systems than the dominant alcohols (like menthol) found in peppermint oil [46]. Carvone has been reported to affect bacterial membrane permeability and to induce the leakage of intracellular components, while carvacrol can strongly disrupt and depolarize the cytoplasmic membrane, resulting in ion imbalance, loss of membrane integrity, and cellular damage. Menthone has likewise been reported to alter membrane integrity and membrane-associated lipid homeostasis, supporting the possibility that both major peppermint EO constituents contribute to its antimicrobial activity. The antimicrobial activity of these major constituents has been associated primarily with perturbation of the bacterial cell envelope, although their relative contribution may differ according to their chemical structure and the bacterial target. Nevertheless, the observed differences in activity cannot be attributed to individual EO constituents based solely on their relative abundance, as both major and minor components may contribute to the overall effect through additive, synergistic, or antagonistic interactions. The observed differences in activity may also be related to the structural characteristics of the major EO constituents. The structural characteristics of major monoterpenoid constituents, including their functional groups and degree of oxygenation, may influence their ability to interact with the bacterial cell envelope and alter membrane permeability. These properties may contribute to the stronger activity observed against planktonic cells, where bacterial cells are directly exposed to the EO constituents. At sub-inhibitory concentrations, interactions with the cell envelope may also modify bacterial surface properties and interfere with the initial adhesion process, which is consistent with the reduction in *E. coli* attachment observed even at 0.5 × MIC. In contrast, established biofilms were less susceptible to EO treatment, most likely because the extracellular polymeric substance matrix can limit the accessibility and diffusion of antimicrobial compounds to cells embedded within the biofilm [19]. Thus, differences in the chemical structures of the major constituents may contribute not only to the direct antibacterial activity of the EOs against planktonic cells but also to their ability to interfere with initial attachment and to reduce established biofilm biomass. However, these relationships should be considered as possible structure–activity associations rather than definitive structure–activity relationships, since the individual compounds were not tested separately in the present study. Thus, the overall antibiofilm efficacy followed the same order as the antibacterial activity determined by MIC and MBC assays (dill > basil > winter savory > peppermint). This consistency suggests that the antimicrobial potency of the tested EOs against planktonic cells may contribute to their ability to reduce established biofilm biomass. This study indicates that essential oils were particularly effective during the early stages of biofilm development. All tested essential oils significantly reduced initial bacterial attachment, even at sub-inhibitory concentrations, demonstrating that biofilm establishment can be impaired before extensive extracellular matrix formation occurs.

This ranking was also observed in the treatment of preformed biofilms. At 2 × MIC after 60 min of exposure, dill EO produced the greatest reduction in biofilm biomass (59.90%), followed by basil EO (50.86%), winter savory EO (47.03%), and peppermint EO (34.00%). Thus, the overall antibiofilm efficacy followed the same order as the antibacterial activity determined by MIC and MBC assays (dill > basil > winter savory > peppermint). This consistency suggests that the antimicrobial potency of the tested EOs against planktonic cells may contribute to their ability to reduce established biofilm biomass. However, the relatively limited reduction observed even under the most effective treatment conditions also demonstrates the increased tolerance of preformed biofilms compared with planktonic cells. The persistence of established biofilm may be partly related to the predominance of the rdar morphotype among the examined isolates, as the curli- and cellulose-associated extracellular matrix characteristic of this phenotype can contribute to biofilm structural stability and increased protection of embedded cells.

The anti-adhesive activity observed at 0.5 × MIC suggests that essential oils can interfere with bacterial surface properties even when concentrations are insufficient to completely inhibit growth. Possible mechanisms include modification of cell surface hydrophobicity, alteration of membrane-associated proteins, interference with adhesin expression, and disruption of early cell-to-surface interactions [47]. At higher concentrations, the stronger inhibition observed at MIC and 2 × MIC likely reflects increased membrane damage and reduced bacterial viability, resulting in impaired colonization capacity. These results highlight the importance of evaluating sub-inhibitory concentrations, as such concentrations may provide valuable information regarding the ability of natural antimicrobials to prevent contamination establishment without necessarily exerting strong bactericidal effects.

In contrast to their strong inhibitory effect on initial attachment, essential oils showed reduced efficacy against mature biofilms. This difference confirms the increased tolerance of established biofilm communities and reflects the protective role of the EPS matrix. Mature biofilms represent highly organized microbial structures in which bacterial cells exhibit altered physiological states, reduced metabolic activity, and increased stress resistance. The EPS matrix limits diffusion of antimicrobial molecules and creates heterogeneous microenvironments that protect deeper bacterial populations from exposure. Consequently, complete biofilm elimination is considerably more difficult than preventing initial attachment [13,14].

The results obtained on lettuce leaves provide additional insight into the practical applicability of essential oils. Although all tested essential oils significantly reduced viable biofilm-associated *E. coli* cells on lettuce surfaces, their effectiveness was consistently lower than that observed on polystyrene surfaces. This difference emphasizes the importance of considering the complexity of real food matrices when evaluating antimicrobial interventions. Unlike homogeneous abiotic surfaces, lettuce leaves contain diverse structural features, including hydrophobic regions, surface irregularities, and protected microsites that facilitate bacterial attachment and reduce antimicrobial accessibility. In addition, the hydrophobic constituents of essential oils may be partially adsorbed or retained by the cuticular waxes and other surface components of lettuce leaves, potentially reducing the fraction of active compounds available for direct interaction with biofilm-associated cells. Therefore, the lower reduction observed on lettuce does not necessarily indicate reduced antimicrobial potential but rather reflects the additional challenges associated with removing biofilms from biological surfaces [45]. Cui et al., 2016 [48] reported greater reductions in *E. coli* O157:H7 biofilms treated with clove essential oil on stainless steel compared with lettuce surfaces, highlighting the influence of surface characteristics on antimicrobial performance.

SEM analysis following essential oil treatment confirmed the quantitative microbiological findings and demonstrated that essential oils affected both the number and structural organization of biofilm-associated cells. Treatment with dill essential oil resulted in fragmentation of bacterial aggregates, disruption of the biofilm matrix, and pronounced morphological alterations of individual cells. Due to their hydrophobic nature, EO constituents can readily partition into bacterial membranes, disrupting membrane integrity, increasing permeability, and promoting the leakage of intracellular constituents, ultimately leading to cell death [34,49]. These microscopic observations provide additional evidence that essential oils act through multiple mechanisms, affecting not only bacterial viability but also the structural stability of established biofilm communities.

The present study provides several important insights into the development and control of *E. coli* biofilms on fresh produce surfaces. A particular novelty of this work is the integrated evaluation of multiple stages of biofilm development, from initial phenotypic characterization and environmental regulation of biofilm formation to bacterial attachment on lettuce leaves and the response of established biofilms to essential oil treatment. While previous studies have primarily focused on the antimicrobial activity of essential oils against planktonic bacteria or biofilms developed on abiotic laboratory surfaces, fewer studies have investigated their activity against *E. coli* biofilms directly formed on lettuce leaves under conditions that better reflect contamination scenarios encountered in the fresh produce chain. The predominance of dill essential oil as the most effective treatment throughout all experimental models represents another relevant finding. Although several essential oils demonstrated antimicrobial activity, dill essential oil consistently showed the greatest reduction in bacterial attachment and established biofilm biomass. This superior performance may provide a basis for selecting specific plant-derived antimicrobials according to their chemical composition and target application.

The statistical analysis further supports the complexity of essential oil-mediated biofilm control by demonstrating that antimicrobial efficacy is determined by the combined effects of essential oil type, concentration, and exposure time. Although concentration represented the dominant factor influencing bacterial inhibition, the significant interactions among essential oil, dose, and treatment duration indicate that antibiofilm activity cannot be explained by a single parameter. These results emphasize the importance of optimizing treatment conditions for each essential oil rather than applying a universal concentration or exposure time.

From an applied perspective, the results obtained in this study are particularly relevant for improving the microbiological safety of leafy vegetables. Lettuce represents a challenging food matrix because its large surface area, irregular structure, and nutrient-rich microenvironments support bacterial attachment and persistence. The demonstrated ability of essential oils to reduce *E. coli* biofilms on lettuce surfaces indicates their potential as natural intervention strategies. Future studies should evaluate the performance of essential oils under realistic processing conditions, including naturally contaminated lettuce, commercial washing systems, different storage temperatures, and longer storage periods. Additional research should also investigate possible synergistic combinations of essential oils or their integration with other non-thermal technologies to improve efficacy while minimizing required concentrations. The findings support the potential application of essential oils as natural antibiofilm agents and provide a foundation for their further development as complementary tools for improving the safety of fresh and minimally processed vegetables.

## 5. Conclusions

The present study demonstrates that *E. coli* persistence on fresh leafy vegetables is closely linked to biofilm establishment, highlighting the importance of targeting bacterial colonization before mature biofilm structures develop. The findings indicate that *E. coli* biofilm formation on fresh produce should be regarded not only as a consequence of bacterial growth but as an environmentally regulated process that can substantially influence persistence on plant surfaces. This emphasizes that environmental conditions encountered during fresh-produce production, handling, and storage should be considered when developing strategies to limit bacterial persistence. The present study demonstrated that the examined *E. coli* isolates exhibited a pronounced biofilm-forming capacity, characterized by the predominance of the rdar morphotype and strong biofilm production. Lower incubation temperature (25 °C) promoted biofilm formation, resulting in greater biofilm biomass and enhanced bacterial attachment to lettuce leaf surfaces compared with 37 °C. Taken together, these findings indicate that controlling the conditions that favor early biofilm establishment may be an important component of strategies aimed at reducing *E. coli* persistence on fresh produce.

The results further demonstrate that spice-derived essential oils have potential as complementary natural interventions for controlling *E. coli* biofilms. Importantly, their greatest practical value may lie in preventing or limiting early bacterial attachment rather than in the complete removal of established biofilms. Their most pronounced effect was observed during initial attachment, whereas established biofilms were considerably more difficult to eliminate. This distinction is important from an application perspective, as essential oils may be more effective as preventive or early-stage interventions than as stand-alone treatments for the removal of mature biofilms. Among the tested essential oils, dill EO exhibited the highest antibacterial, anti-adhesion, and antibiofilm activity, followed by basil, winter savory, and peppermint EOs. These findings suggest that the timing of application may be as important as the antimicrobial potency of the essential oil when developing an effective intervention. The reduced efficacy observed on lettuce leaves compared with polystyrene surfaces further emphasizes the importance of evaluating antibiofilm agents under conditions that closely resemble real food matrices. Thus, laboratory efficacy alone should not be considered sufficient to predict the performance of essential oils on fresh produce, where surface structure and interactions with the plant matrix can substantially influence antimicrobial accessibility.

SEM analysis confirmed the quantitative findings by revealing disruption of biofilm architecture, fragmentation of bacterial aggregates, and structural damage to bacterial cells following EO treatment, supporting membrane disruption as one of the principal mechanisms underlying their antibiofilm activity.

Overall, this study demonstrates that spice-derived EOs, particularly dill essential oil, represent promising natural agents for controlling *E. coli* biofilms on fresh produce. Rather than replacing conventional sanitation, their most realistic role may be as complementary interventions integrated into strategies aimed at preventing early bacterial attachment and biofilm establishment. Nevertheless, further studies under industrial processing conditions are needed to optimize application methods, evaluate sensory effects and product quality, assess long-term efficacy on naturally contaminated produce, and determine their feasibility for large-scale commercial use. Future research should also determine how essential oils can be integrated with existing non-thermal or washing technologies to improve efficacy while maintaining the sensory and quality characteristics of fresh produce. Such validation will be essential for determining whether the promising antibiofilm activity observed in this study can be translated into a practical intervention for improving the microbiological safety of leafy vegetables.

## Figures and Tables

**Table 1:** Antimicrobial activity of the essential oils against *E. coli*.

Essential Oils	MIC (µL/mL)	MBC (µL/mL)
*Anethum graveolens*	3.55 ± 1.78	3.55 ± 0.00
*Mentha piperita*	14.20 ± 0.00	14.20 ± 0.00
*Ocimum basilicum*	7.10 ± 0.00	7.10 ± 0.00
*Satureja montana*	7.10 ± 1.78	7.10 ± 1.78

Values represent the mean value ± SD obtained in three independent replicates. MIC—minimum inhibitory concentration, MBC—minimum bactericidal concentration.

## Data Availability

The original contributions presented in this study are included in the article. Further inquiries can be directed to the corresponding authors.

## References

[1] Shi M., Gu J., Wu H., Rauf A., Emran T.B., Khan Z., Mitra S., Aljohani A.S.M., Alhumaydhi F.A., Al-Awthan Y.S. (2022). Phytochemicals, nutrition, metabolism, bioavailability, and health benefits in lettuce-A comprehensive review. Antioxidants.

[2] Santos M.I., Grácio M., Silva M.C., Pedroso L., Lima A. (2023). One health perspectives on food safety in minimally processed vegetables and fruits: From farm to fork. Microorganisms.

[3] Bulut E., Murphy S.I., Strawn L.K., Danyluk M.D., Wiedmann M., Ivanek R. (2025). Risk assessment of *Escherichia coli* O157:H7 along the farm-to-fork fresh-cut romaine lettuce supply chain. Sci. Rep..

[4] Ölmez H. (2016). Foodborne pathogenic bacteria in fresh-cut vegetables and fruits. Food Hygiene and Toxicology in Ready-To-Eat Foods.

[5] Carstens C.K., Salazar J.K., Darko C. (2019). Multistate outbreaks of foodborne illness associated with leafy greens: A review of epidemiological evidence and pathogen transmission pathways. Food Control.

[6] Centers for Disease Control and Prevention (CDC) (2023). Foodborne Outbreak Investigation and Surveillance Data: Leafy Greens and Other Produce-Associated Outbreaks.

[7] Yaron S., Römling U. (2014). Biofilm formation by enteric pathogens and its role in plant colonization and persistence. Microb. Biotechnol..

[8] Balagopal A., Kothandapani S., Prathapkumar H.S. (2017). Study on *E. coli* and *Salmonella* biofilms from fresh fruits and vegetables. J. Food Sci. Technol..

[9] Uruén C., Chopo-Escuin G., Tommassen J., Mainar-Jaime R.C., Arenas J. (2020). Biofilms as promoters of bacterial antibiotic resistance and tolerance. Antibiotics.

[10] Rossi C., Chaves-López C., Serio A., Casaccia M., Maggio F., Paparella A. (2022). Effectiveness and mechanisms of essential oils for biofilm control on food-contact surfaces: An updated review. Crit. Rev. Food Sci. Nutr..

[11] Jacob C., Melotto M. (2020). Human pathogen colonization of lettuce dependent upon plant genotype and defense response activation. Front. Plant Sci..

[12] Safwa M.D., Ahmed T., Talukder S., Sarkar A., Rana M.R. (2024). Applications of non-thermal technologies in food processing Industries-A review. J. Agric. Food Res..

[13] Tomičić R., Čabela M., Tomičić Z., Čabarkapa I., Kocić-Tanackov S., Raspor P. (2025). ZnO nanoparticles enhance the efficiency of sodium hypochlorite disinfectant in reducing the adhesion of pathogenic bacteria to stainless steel surfaces. Food Microbiol..

[14] Tomičić R., Tomičić Z., Nićetin M., Knežević V., Kocić-Tanackov S., Raspor P. (2023). Food grade disinfectants based on hydrogen peroxide/peracetic acid and sodium hypochlorite interfere with the adhesion of *Escherichia coli*, *Pseudomonas aeruginosa*, *Staphylococcus aureus* and *Listeria monocytogenes* to stainless steel of differing surface roughness. Biofouling.

[15] Elbehiry A., Marzouk E., Alzaben F., Almuaither A., Abead B., Alamri M., Almuzaini A.M., Abu-Okail A. (2025). Emerging technologies and integrated strategies for microbial detection and control in fresh produce. Microorganisms.

[16] Kocić-Tanackov S., Dimić G., Mojović L., Pejin J., Tanackov I. (2014). Effect of caraway, basil, and oregano extracts and their binary mixtures on fungi in growth medium and on shredded cabbage. LWT—Food Sci. Technol..

[17] Laranjo M., Fernández-Léon A.M., Potes M.E., Agulheiro-Santos A.C., Elias M., Méndez-Vilas A. (2017). Use of essential oils in food preservation. Antimicrobial Research: Novel Bioknowledge and Educational Programs.

[18] Bukvicki D., D’Alessandro M., Rossi S., Siroli L., Gottardi D., Braschi G., Patrignani F., Lanciotti R. (2023). Essential oils and their combination with lactic acid bacteria and bacteriocins to improve the safety and dhelf life of foods: A Review. Foods.

[19] Yammine J., Chihib N.E., Gharsallaoui A., Dumas E., Ismail A., Karam L. (2022). Essential oils and their active components applied as: Free, encapsulated and in hurdle technology to fight microbial contaminations. A review. Heliyon.

[20] Panda S.K., Buroni S., Swain S.S., Bonacorsi A., da Fonseca Amorim E.A., Kulshrestha M., da Silva L.C.N., Tiwari V. (2022). Recent advances to combat ESKAPE pathogens with special reference to essential oils. Front. Microbiol..

[21] Solomon E.B., Niemira B.A., Sapers G.M., Annous B.A. (2001). Biofilm formation, cellulose production, curli biosynthesis by *Salmonella* originating from produce, animal, and clinical sources. J. Food Prot..

[22] Lu Y., Dong H., Chen S., Chen Y., Peng D., Liu X. (2011). Characterization of biofilm formation by *Salmonella enterica* serovar Pullorum strains. Afr. J. Microbiol. Res..

[23] Bokranz W., Wang X., Tschäpe H., Römling U. (2025). Expression of cellulose and curli fimbriae by *Escherichia coli* isolated from the gastrointestinal tract. J. Med. Microbiol..

[24] Römling U. (2005). Characterization of the rdar morphotype, a multicellular behavior in *Enterobacteriaceae*. Cell Mol. Life Sci..

[25] Council of Europe (2004). European Pharmacopoeia.

[26] Varga A., Kocić-Tanackov S., Čabarkapa I., Aćimović M., Tomičić Z. (2019). Chemical composition and antibacterial activity of spice essential oils against *Escherichia coli* and *Salmonella* Typhimurium. Arch. Fur. Liturg..

[27] (2012). Methods for Dilution Antimicrobial Susceptibility Tests for Bacteria That Grow Aerobically.

[28] Stepanović S., Vuković D., Dakić I., Savić B., Švabić-Vlahović M. (2000). A modified microtiter-plate test for quantification of staphylococcal biofilm formation. J. Microbiol. Methods.

[29] Correa M.S., Schwambach J., Mann M.B., Frazzon J., Frazzon A.P.G. (2019). Antimicrobial and antibiofilm activity of the essential oil from dried leaves of *Eucalyptus staigeriana*. Pharmacology.

[30] Berger C.N., Shaw R.K., Brown D.J., Mather H., Clare S., Dougan G., Pallen M.J., Frankel G. (2009). Interaction of *Salmonella enterica* with basil and other salad leaves. ISME J..

[31] Patel J., Sharma M. (2010). Differences in attachment of *Salmonella enterica* serovars to cabbage and lettuce leaves. Int. J. Food Microbiol..

[32] Trevisan D.A.C., Silva A.F., Negri M., Abreu Filho B.A., Machinsky Junior M., Patussi E.V., Campanerut-Sá P.A.Z., Mikcha J.M.G. (2018). Antibacterial and antibiofilm activity of carvacrol against *Salmonella enterica* serotype Typhimurium. Braz. J. Pharm. Sci..

[33] Pathan A.K., Bond J., Gaskin R.E. (2008). Sample preparation for scanning electron microscopy of plant surfaces-horses for courses. Micron.

[34] Miladi H., Zmantar T., Kouidhi B., Mohammed Y., Al Quarashi Y.M.A., Bakhrouf A., Chaabouni Y., Mahdouani K., Chaieb K. (2017). Synergistic effect of eugenol, carvacrol, thymol, p-cymene and γ-terpinene on inhibition of drug resistance and biofilm formation of oral bacteria. Microb. Pathog..

[35] Tomičić Z., Čabarkapa I., Šarić L., Džomba M., Tomičić R. (2023). Germicidal efficacy of disinfectant based on sodium hypochlorite and essential oils. J. Food Nutr. Res..

[36] Meenu M., Padhan B., Patel M., Patel R., Xu B. (2023). Antibacterial activity of essential oils from different parts of plants against *Salmonella* and *Listeria* spp.. Food Chem..

[37] Simm R., Ahmad I., Rhen M., Le Guyon S., Römling U. (2014). Regulation of biofilm formation in Salmonella enterica serovar Typhimurium. Future Med..

[38] Milanov D., Prunić B., Velhner M., Todorović D., Polaček V. (2015). Investigation of biofilm formation and phylogenetic typing of *Escherichia coli* strains isolated from milk of cows with mastitis. Acta Vet..

[39] Römling U., Rohde M., Olsen A., Normak S., Reinköster J. (2000). AgfD the checkpoint of multicellular and aggregative behavior in *Salmonella* Typhimurium regulates at least two independent pathways. Mol. Microbiol..

[40] Solano C., García B., Valle J., Berasain C., Ghigo J.M., Gamazo C., Lasa I. (2002). Genetic analysis of *Salmonella enteritidis* biofilm formation: Critical role of cellulose. Mol. Microbiol..

[41] Römling U., Kjelleberg S., Normark S., Nyman L. (2013). Microbial biofilm formation: A need to act. J. Intern. Med..

[42] Kroupitski Y., Pinto R., Brandl M.T., Belausov E., Sela S. (2009). Interactions of *Salmonella enterica* with lettuce leaves. J. Appl. Microbiol..

[43] Lapidot A., Yaron S. (2009). Transfer of *Salmonella enterica* serovar Typhimurium from contaminated irrigation water to parsley is dependent on curli and cellulose, the biofilm matrix components. J. Food Prot..

[44] Bridier A., Briandet R., Thomas V., Dubois-Brissonnet F. (2015). Resistance of bacterial biofilms to disinfectants: A review. Biofouling.

[45] Hunter P.J., Shaw R.K., Berger C.N., Frankel G., Pink D., Hand P. (2015). Older leaves of lettuce (*Lactuca* spp.) support higher levels of *Salmonella enterica* ser. Senftenberg attachment and show greater variation between plant accessions than younger leaves. FEMS Microbiol. Lett..

[46] Jannesar N., Bassiri A., Ghavami M., Chenarbon H.A., Tarzi B.G. (2024). Investigation of physicochemical and antibacterial properties of dill (*Anethum graveolens* L.) microencapsulated essential oil using fluidized bed method. Food Chem. X.

[47] Borges A., Saavedra M.J., Simões M. (2012). The activity of ferulic and gallic acids in biofilm prevention and control of pathogenic bacteria. Biofouling.

[48] Cui H., Ma C., Liu L. (2016). Synergetic antibacterial efficacy of cold nitrogen plasma and clove oil against *Escherichia coli* O157:H7 biofilms on lettuce. Food Control.

[49] Diao W.R., Hu Q.P., Zhang H., Xu J.G. (2014). Chemical composition, antibacterial activity and mechanism of action of essential oil from seeds of fennel (*Foeniculum vulgare* Mill.). Food Control.

